# PRMT1-mediated methylation of UBE2m promoting calcium oxalate crystal-induced kidney injury by inhibiting fatty acid metabolism

**DOI:** 10.1038/s41419-025-07888-3

**Published:** 2025-07-31

**Authors:** Tianhui Yuan, Zehua Ye, Shuqin Mei, Miao Zhang, Ming Wu, Fangyou Lin, Weimin Yu, Wei Li, Xiangjun Zhou, Fan Cheng

**Affiliations:** 1 Department of Urology, Renmin Hospital of Wuhan University, Wuhan, China; 2 Department of Nephrology, Shanghai Changzheng Hospital, Naval Medical University, Shanghai, China; 3 Department of Nephrology, Shuguang Hospital Affiliated to Shanghai University of Traditional Chinese Medicine, Shanghai, China; 4 Department of Anesthesiology, Renmin Hospital of Wuhan University, Wuhan, China

**Keywords:** Cell signalling, Neddylation, Methylation, Ubiquitylation, Renal calculi

## Abstract

Calcium oxalate (CaOx) is the most common type of kidney stone, and its crystal deposition can induce oxidative stress, inflammatory responses, and cell death. This further aggravates kidney structural and functional damage, which in turn, promotes kidney stone recurrence, forming a vicious cycle of repeated stone formation and renal injury. Therefore, identifying precise and effective therapeutic targets is crucial to prevent the damage and inflammation caused by kidney stones. Protein arginine methyltransferase 1 (PRMT1) is a well-known epigenetic regulatory enzyme involved in renal metabolic reprogramming. However, the role of PRMT1-mediated arginine methylation in kidney stone-induced renal injury remains unclear. In this study, mice with specific deletion or overexpression of PRMT1 in tubular epithelial cells were developed, and a CaOx crystal-induced kidney injury mouse model was established. Single-cell RNA-sequencing, metabolomic, proteomic, and transcriptomic analyses, together with immunoprecipitation, mass spectrometry, GST-pulldown assays, oxygen consumption rate assays, and other methods, were used to reveal the mechanism of PRMT1 in renal injury caused by CaOx crystals. Specifically, PRMT1 enhanced the protein function of UBE2m through arginine methylation at R169, and increased the neddylation level and protein stability of NEDD4, thereby inducing PPARγ ubiquitination. Increased PPARγ degradation inhibited downstream fatty acid metabolism, leading to renal lipid accumulation, disrupted energy metabolism, and impaired kidney function. These findings provide a novel potential therapeutic target for CaOx kidney stones.

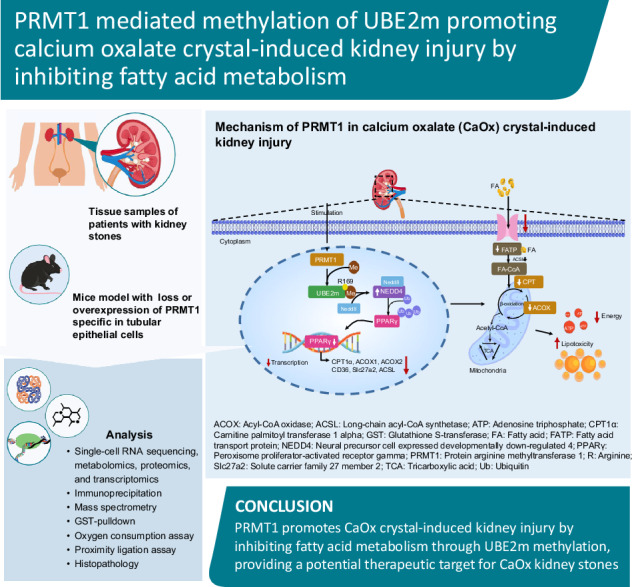

## Introduction

Nephrolithiasis, commonly known as kidney stone disease, is a prevalent and recurrent urological condition with a recurrence rate of up to 50% within the first five years [1, 2]. It is characterized by inflammation and tissue injury, the driving factors in stone re-formation [3, 4]. Repeated stone formation further aggravates tissue injury, thus forming a vicious circle [5]. Surgical crushing alone does not resolve the increasing prevalence of kidney stones (KS). While some therapeutic approaches have shown promise in limiting renal injury and fibrosis in clinical trials [2, 6], the overall clinical efficacy remains limited. A promising strategy is necessary to address the renal damage and crystal aggregation inherent to KS.

A critical aspect of renal stone pathogenesis and subsequent damage involves metabolic alterations, particularly in fatty acid metabolism [7, 8]. As the primary energy source for tubular epithelial cells (TECs), fatty acid oxidation (FAO) supports critical cellular functions under normal conditions. However, alterations in fatty acid metabolism during renal injury contribute to metabolic reprogramming and oxidative stress, ultimately promoting renal fibrosis [7, 9]. Recent studies have highlighted the intricate interplay between fatty acid metabolism and renal disease progression, revealing potential therapeutic targets [8, 10].

Concurrently, protein arginine methylation, a post-translational modification facilitated by the family of protein arginine methyltransferases, has been recognized as an essential regulator of cellular functions in multiple organs [11, 12]. Among the PRMT family, PRMT1 stands out due to its broad substrate specificity and abundance in various cell types, including renal cells [11, 13]. PRMT1 catalyzes the mono- and asymmetric dimethylation of arginine residues, thereby influencing protein function, stability, and subcellular localization [14, 15]. Its expression patterns and functional roles in the kidney are well-documented, providing a solid foundation for further investigation [16]. However, how PRMT1 influences CaOx stone-induced kidney injury remains largely unknown. Recent studies have implicated PRMT1 in regulating fatty acid metabolism, suggesting that it might be a pivotal node in the complex interplay between metabolic reprogramming and renal injury [17–19]. Perturbations in PRMT1 activity affect the expression and function of enzymes involved in FAO, thereby modulating cellular energy metabolism [20]. Therefore, targeting PRMT1 holds promise as a novel therapeutic strategy, particularly in kidney stone disease, in which metabolic derangement and inflammation are prevalent.

In this study, we confirmed that PRMT1 enhanced the neddylation function of ubiquitin-conjugating enzymes E2M (UBE2m) through arginine methylation at R169, increasing the neddylation level and protein stability of NEDD4. This promoted Peroxisome proliferator activated receptor γ (PPARγ) ubiquitination and degradation, leading to the inhibition of fatty acid metabolism. Our findings elucidate the underlying mechanisms and highlight potential therapeutic targets for CaOx crystal-induced kidney injury.

## Methods

### Human samples study

Samples were obtained from eight patients with nonfunctioning kidneys due to KS undergoing nephrectomy at Renmin Hospital of Wuhan University (mean age 49.1 ± 8.0 years; five males and three females) (Table S1). The stones were identified as CaOx monohydrate or dihydrate using stone composition analysis (Fig. S1A). Eight normal tissue samples were obtained from the adjacent non-tumorous tissues of patients who had undergone radical renal resection (mean age 59.9 ± 6.1 years; five males and three females) as the normal controls (NC) (Table S1). The study protocol adhered to the Declaration of Helsinki and was approved by the review board of Renmin Hospital of Wuhan University (WDRY2021-KS047). Informed consent was obtained from all participants prior to sample collection.

### Single-cell RNA sequencing

Human kidney samples were prepared as single-cell suspensions according to the manufacturer’s instructions. The single-cell library was established according to the BD Rhapsody Single-Cell 3ʹ Whole Transcriptome (WTA) Library Construction Standardization Manual. After library preparation and quality control, double-end sequencing was performed on the Illumina Nova Seq6000 sequencing platform in 2 × 150 bp mode. Raw BCL files were assembled using Illumina’s bcl2fastq converter to obtain raw fastq data, and high-quality clean reads were obtained after data filtering through primary quality control. Subsequently, the BD Rhapsody analysis process was used for quality control and filtering of data, identification of cell ID and molecule ID, mapping of reads to the reference genome, quantification of gene expression and identification of cells, and, finally, generation of the original expression matrix. Sctype software was used for automatic annotation, followed by manual inspection and correction for cell type annotations. The FindMarkers and FindAllMarkers functions, with default parameters set, were used to identify differentially expressed genes between each subgroup.

### Animal experiments

The Animal Care and Use Committee of Renmin Hospital of Wuhan University approved all animal experiments (WDRM 20200604), which complied with the Guide for the Care and Use of Laboratory Animals (NIH Publication No. 85-23, revised 1996). Transgenic mice expressing Cre-recombinase under the cadherin 16 (Cdh16) promoter were purchased from the Jackson Laboratory (JAX 012237) [21]. The Cdh16 (also known as Ksp-cadherin) promoter was activated in the TECs of the kidneys [22, 23]. PRMT1 floxed mice (C57BL/6J background, PRMT1fl/fl) were obtained from Shanghai Model Organisms. Renal tubular epithelial-specific deletion of PRMT1 mice were generated by crossing floxed PRMT1 mice with Cdh16-Cre± mice and wild-type littermates were used as controls. Genotyping was performed using agarose gel electrophoresis. PRMT1 identification: Homozygotes = 330 bp; Heterozygotes = 330 bp and 273 bp; Wild-type allele = 273 bp (Fig. S2H). Cdh16cre identification: Cre primers amplified 400 bp bands indicating Cdh16Cre positive (Fig. S2I).

PRMT1 was specifically overexpressed in TECs in mice by injecting recombinant adeno-associated virus 2 (rAAV9) (Genomeditech, Shanghai, China) into the bilateral renal parenchyma using a microliter syringe. The injection involved inserting the needle from the lower pole to the upper pole of the kidney and gradually withdrawing it after administering the rAAV9 solution. Western blotting (WB) and EGFP fluorescence were used to detect transgene expression levels and distribution after 4 weeks (Fig S2C–E). rAAV9-PRMT1 was developed by cloning the target gene PRMT1 into an AAV serotype 9 vector containing the kidney-specific promoter KSPC (GPAAV-KSP0.3k-Mouse_Prmt1-T2A-eGFP-WPRE) (Fig. S2A).

All the mice (6–8 weeks) were maintained in a pathogen-free environment with ad libitum access to food and water, and a suitable temperature range of 20–25 °C and 50–65% relative humidity. Animals were randomly assigned to experimental groups using a computer-generated sequence. Outcome assessors were blinded to group allocation. Each mouse was injected intraperitoneally with glyoxylate (Gly, 100 mg/kg/d) for 10 consecutive days to establish a hyperoxaluria mouse model. Mice within the control group were subjected to intraperitoneal administration of the same volume of saline. Mice received atorvastatin at a dose of 20 mg/kg/day via oral gavage.

Sample size estimation was guided by previously published studies using similar models. For in vivo studies, a minimum of five mice per group was selected, which has been shown to be sufficient to detect biologically and statistically significant differences in similar experiments. For in vitro experiments, at least three independent replicates were performed to ensure reproducibility.

Mice with signs of unrelated illness, abnormal baseline renal function, or technical failure during surgery/crystal injection were excluded from analysis. For in vitro studies, data from wells with contamination, low cell viability (<80%), or technical artifacts were excluded.

### Reagents

The following is a detailed description of the reagents used. Antibodies against PRMT1 (#2449, 1:1000 for WB, 1:100 for IF or IHC, 1:50 for IP), monomethyl arginine (#8015, 1:1000 for WB), FATP2 (#78771, 1:1000 for WB), PPARγ (#2435, 1:1000 for WB, 1:30 for IP), and NEDD8 (#2745, 1:1000 for WB) were purchased from Cell Signaling Technology. Anti-CD36 (ab252922, 1:1000 for WB) and Anti-NEDD4 (ab245522, 1:1000 for WB, 5 µg/mg for IP) were purchased from Abcam. Antibodies against UBE2m (14520-1-AP, 1:1000 for WB, 1:100 for IF or IHC, 1:50 for IP), CPT1a (15184-1-AP, 1:8000 for WB), ACOX1 (10957-1-AP, 1:2000 for WB), FlAG (20543-1-AP, 1:30,000 for WB, 1 µg/mg for IP), His (66005-1-Ig, 1:10,000 for WB, 1 µg/mg for IP), GST (66001-2-Ig, 1:8000 for WB, 1 µg/mg for IP), HA (51064-2-AP, 1:8000 for WB, 1 µg/mg for IP), and Myc (60003-2-Ig, 1:4000 for WB, 1 µg/mg for IP) were purchased from Proteintech. Site-specific antibody meUBE2m (R169me1), 1:500 by immunoblotting and 1:50 by IHC staining, recognizing UBE2m monomethyl-R169 was produced using the synthetic peptide sequence EQNVQRSM-R(me1)-GGYI (PTM Bio, Hangzhou, China). Anti-Lotus Tetragonolobus Lectin (LTL) (FL-1321, VECTOR Laboratories), anti-peanut agglutinin (PNA) (FL-1071, VECTOR Laboratories), and anti-Dolichos Biflorus Agglutinin (DBA) (FL1031, VECTOR Laboratories) were purchased from VECTOR. Pevonedistat (MLN4924) (HY-70062) were purchased from MedChem Express. Glyoxylate (Gly; G10601) was purchased from Sigma-Aldrich (St. Louis, MO, USA).

### Cell culture

HK-2 and HEK293T cells were maintained in Dulbecco’s Modified Eagle Medium (DMEM) supplemented with 10% fetal bovine serum (FBS) and 1% penicillin-streptomycin at 37 °C in a 5% CO_2_ incubator. All cells from the Chinese Academy of Sciences Typical Culture Repository were recently identified using short tandem repeat fingerprinting and tested negative for mycoplasma. Cells were stimulated with 100 μg/mL of calcium oxalate monohydrate (COM) for 24 h to construct the in vitro cell model based on previous studies. In addition, cells were incubated with pevonedistat (1 μg/mL) to determine the effect of neddylation of NEDD4 [22, 24, 25].

### Comprehensive fatty acyl-CoA assay

A comprehensive analysis of fatty acyl-CoA in the renal cortex was conducted on five renal tissue samples from each group. A 300 μL extract containing isopropyl alcohol, 50 mM KH2PO4, and 50 mg/mL BSA (25:25:1 v/v/v), acidified with glacial acetic acid, was added to the cells. The internal standard 19:0-CoA was then introduced and incubated at 4 °C at 1500 rpm for 1 h to extract lipids. After incubation, 300 μL petroleum ether was added and centrifuged at 4 °C at 12,000 rpm for 2 min to remove the upper phase; this extraction step was repeated twice with petroleum ether. Subsequently, 5 μL saturated ammonium sulfate followed by 600 μL chloroform:methanol (1:2 v/v) were added to the final lower phase. The samples were incubated in a constant temperature mixer at 25 °C and stirred at 450 rpm for 20 min before being centrifuged at 4 °C at 12,000 rpm for 5 min. The supernatant containing long-chain acyl-CoAs was transferred to a new Eppendorf tube for drying using a SpeedVac (Genevac) in OH mode. An additional extraction of the remaining precipitate with trichloroacetic acid (1 mL) was performed to enhance the recovery of polar short-chain acyl-CoAs. As previously described, this acid extract underwent solid-phase purification using an Oasis HLB SPE column (Waters). The purified extract containing polar CoAs was also dried in a SpeedVac in OH mode. Finally, both extracts were combined and suspended in a solution of methanol:water (9:1 v/v) containing 0.05% acetic acid prior to analysis using a Shimadzu UPLC-SCIEX QTRAP6500 Plus.

### Isolation of mouse primary tubule epithelial cells (PTECs)

Mouse PTECs were isolated using a modified protocol, as described previously [26, 27]. Briefly, the renal cortex was separated with a sterile scalpel and cut into 1 mm^3^ pieces, transferred to collagenase solution (DMEM/F-12 medium containing 0.1% collagenase IV and 0.1% bovine serum albumin), and digested at 37 °C for 30 min. Then, the supernatant was filtered through two nylon sieves with pore sizes of 100 and 40 μm, respectively. The proximal tubule (PT) fragments in the 40 μm sieve were backwashed with warm medium. PTs present in the medium were centrifuged at 170 × *g* for 5 min, washed, and resuspended in DMEM/F-12 medium supplemented with 10% FBS and cultured at 5% CO_2_ at 37 °C. For treatment, TECs were cultured with or without COM.

### shRNA knockdown and CRISPR/Cas9 knockout cells

shRNA sequences targeting PRMT1 (CCTGGTGGCCTACTTCAACAT), UBE2m (GCGGATCCAGAAGGACATAAA), or NEDD4 (CGCCTTGACTTACCTCCATAT) were cloned into PGMLV-SC5 vectors to generate PRMT1, UBE2m, or NEDD4 stable knockdown (KD) cell lines. Previous studies have shown that a two-plasmid packaging system can be used to create retroviruses. The cells were treated with puromycin (1 μg/mL) for 1 week after retrovirus infection.

The UBE2m knockout (KO) cell line was produced using the CRISPR-Cas9 genome editing technique. Cells were infected with the lentivirus containing the specific gRNA targeting UBE2m (5ʹ-CACCCCAACATTGACCTCG-3ʹ) that was cloned into the LentiCRISPRv2-puro vector, then selected with puromycin (1 μg/mL) for 1 week. Re-expression of UBE2m (wild type (WT) or R169K) in UBE2m KO cells was achieved by using the pQCHIX-hygro vector. A two-plasmid packaging system was used to create the retrovirus, which was then transfected into UBE2m KO cells. The cells were then given a week-long treatment of hygromycin (200 μg/mL).

### Transcriptome RNA sequencing and bioinformatics analysis

From the aforementioned animal studies, six kidney tissues (*n* = 3) from the NC group and three from the Gly group (*n* = 3) were selected for transcriptome RNA sequencing. Total RNA was extracted using TRIzol reagent (15596026CN, Invitrogen, USA) in accordance with the manufacturer’s instructions. The BGI Group (Shenzhen, China) was responsible for the RNA sequencing and data analysis. The Gly and NC groups were compared using the DESeq2 [28] R software package to identify the differentially expressed genes (DEGs) (adj. *p* < 0.05 and |fold change| > 1.2). The “ggplot2” R software package was used to construct volcano plots for data visualization. Kyoto Encyclopedia of Genes and Genomes (KEGG) and Gene Ontology (GO) enrichment analysis was conducted using the R “ClusterProfiler” package to investigate the roles of DEGs. Statistical significance was set at *p* < 0.05.

### Proteome sequencing and bioinformatics analysis

From the aforementioned animal studies, six kidney tissues were selected for proteome sequencing: three from the Gly group (*n* = 3) and three from the Conditional Knockout (CKO) group (*n* = 3). A cracking solution (8 M Urea/100 mM Tris-HCl, pH 8.5) was added to the sample, and the supernatant was obtained by centrifugation after ultrasonic crushing. The protein concentration was measured using the BCA method, and was supplemented with TCEP and CAA at 37 °C for 1 h for reductive alkylation. Urea was diluted to <2 M, using tryptic digestion (1:50) at 37 °C overnight. TFA was terminated the next day, and the supernatant was desalted using SDB-RPS, vacuum-drained, and stored at −20 °C. UltiMate 3000 RSLCnano and TimsTOF Pro mass spectrometers were used for simple detection. Peptides were captured, separated on a C18 column, and eluted using a mobile phase gradient. Data were collected in diaPASEF mode to optimize the voltage, scan range, and ion mobility settings to ensure efficient mass spectrometry. The results of DIA-NN were processed using R, the quantitative signals were converted by log2 for statistical analysis, the proteins with high missing values were removed, and the missing data were interpolated. An unpaired t-test was used to identify differentially expressed proteins (*p* < 0.05, multiplex change >1.5 or <1/1.5). Cluster analysis was performed based on GO, KEGG, and other functional databases, and Fisher’s test was used to screen for significantly enriched items. Gene set enrichment analysis (GSEA) identified significantly changed feature gene sets, and the STRING database was used to construct a protein interaction network (comprehensive score >0.4).

### Untargeted metabolomics

Metabolomics analysis was performed on five renal tissue samples from each group. The frozen tissues were weighed, and then 1.5 mL 80% methanol solution containing 4 μg/mL 2-chloro-L-phenylalanine was homogenized for 2 min. The homogenate was then centrifuged at 13,000 rpm at 4 °C for 15 min. A total of 10 μL of each sample was mixed with the quality control samples. The prepared supernatant was subjected to liquid chromatography-mass spectrometry (LC-MS)/MS.

A Waters 2777c UPLC system (Waters, USA) coupled with a Q Exactive HF high-resolution mass spectrometer (Thermo Fisher Scientific) was utilized for the isolation and identification of metabolites. Chromatographic separation was performed using a Waters ACQUITY UPLC BEH C18 column (1.7 μm, 2.1 mm × 100 mm, Waters, USA), with the column temperature maintained at 45 °C. The Q Exactive HF (Thermo Fisher Scientific) facilitated the acquisition of both primary and secondary MS data. Following the importation of offline MS data into Compound Discoverer 3.3 (ThermoFisher Scientific), analysis using the BGI Metabolome Database (BMDB), mzCloud database, and ChemSpider online database was conducted to generate a comprehensive data matrix that included details, such as metabolite peak areas and identification results. The dataset underwent further processing involving quality control measures, differential analysis, and enrichment analysis.

### Immunoprecipitation

The Pierce Classic Magnetic IP/Co-IP Kit (88804, Thermo Fisher Scientific) was used for immunoprecipitation. In summary, protein lysates were prepared using Pierce IP Lysis/Wash Buffer containing protease and phosphatase inhibitor mixtures (Biosharp) and incubated with IP antibody overnight at 4 °C. The antigen–antibody complex was bound to protein A/G magnetic beads for 1 h at 25 °C, washed with IP Lysis/Wash buffer and purified water, eluted with the antigen/antibody complex, and analyzed using standard immunoblotting protocols.

### Mass spectrometry (MS)

The cellular PRMT1-binding protein was identified using co-immunoprecipitation and analyzed using MS. Briefly, FLAG beads were used to capture IP HK-2 cell lysates that stably expressed FLAG-PRMT1. Immunoprecipitates were isolated using SDS-PAGE. The SDS-PAGE gel was processed according to the instructions for the silver dyeing kit (P0017S, Beyotime, China). All samples (FLAG-PRMT1-binding protein) were analyzed on an UltiMate 3000 RSLCnano system coupled online with Q Exactive HF mass spectrometer through a Nanospray Flex ion source (Thermo Fisher Scientific). MaxQuant was utilized for MS raw data analysis and the Andromeda database search algorithm for protein identification. Proteins with a fold change >4 between bait IP and control were identified as interactors of the bait protein.

His beads, SDS-PAGE, and Coomassie Brilliant Blue (CBB) staining were used to determine the potential UBE2m methylation site expressed in HK-2 cells. His-UBE2m protein gel strip was digested in the gel using α-lytic protease (MilliporeSigma, A6362) and then analyzed using LC-MS/MS in collaboration with SpecAlly (Wuhan, China). A Dionex Ultimate 3000 high-performance liquid chromatography system and Q Exactive quadrupole orbitrap mass spectrometer (Thermo Fisher) were used to analyze the UBE2m-derived peptides. The gradient started with 5% solvent B (0.1% formic acid, 100% acetonitrile) and increased to 32% over 70 min. The DDA mode was used for mass spectrum acquisition. The range was set to 375–1600 m/z for a full MS scan. MS/MS data were processed using Proteome Discoverer 2.5 (Thermo Fisher, RRID: SCR_014477). Trypsin was used to allow for two missing cutting sites. The mass tolerance for the MS/MS analysis was 20 ppm for the precursor and 0.05 Da for the fragment. The fixed modification included the aminomethylation of cysteine residues (57.0215 Da). Variable modifications included mono-methylation of arginine residues (14.01565 Da), dimethylation (28.0313 Da), and oxidation of methionine residues (15.9949 Da).

### Plasmid constructions and site-directed mutagenesis

The PRMT1, UBE2m, NEDD4, PPARγ, and Ub expression vectors were constructed as follows. The GATEWAY transformation system (Thermo Fisher Scientific) was used to convert the inserted fragment into an n-terminal 3xFLAG, His, HA, GST, or Myc labeled destination vector (for expression in mammalian cells). A Fast Site-directed Mutagenesis Kit (FM111, TransGen Biotech) was used to generate point mutations in UBE2m (R33K, R116K, R157K, R158K, R166K, R169K, and R180K). The cells were transfected with empty plasmid vectors or plasmids using Lipofectamine 2000 (Invitrogen, Waltham, MA, USA) according to the manufacturer’s instructions.

### Purification of human recombinant PRMT1 and UBE2m

Human recombinant PRMT1 and UBE2m proteins were produced using the *Escherichia coli* expression system. The protein sequences are shown in Table S2. Each PCR fragment was subcloned into the bacterial expression vector pGEX-6P (GE Healthcare). The plasmids were transformed into *E. coli* BL21. Subsequently, 0.1 mM IPTG was added to the transfected cells, which was then cultured with 1 L LB medium for 16 h at 27 °C. After collection via centrifugation, the cells were incubated in PBS on ice for 10 min. The lysates were sonicated, separated via centrifugation, and loaded onto a GSTrap FF column (17513001, GE Healthcare). After washing twice with PBS, the eluate (50 mM Tris-HCl (pH 7.8) and 10 mM glutathione) was dialyzed with PBS. The purification efficiency of each preparation was determined using CBB staining.

### GST-pulldown

The aforementioned technique was used to obtain fusion proteins. The GST-PRMT1 fusion protein or GST protein was mixed with His-UBE2m fusion protein overnight under gentle rotation at 4 °C and then incubated with GST beads (78602, Thermo Fisher Scientific) at 4 °C for 3 h. The beads were then subsequently rinsed with IP Lysis/Wash buffer, heated in SDS loading buffer, and analyzed through immunoblotting.

### In vitro methylation assays

The aforementioned technique was used to obtain fusion proteins. A total of 5 μg His-UBE2m and 5 μg GST-PRMT1 were added into a standard 50-μL reaction mixture containing 5 μl S-adenosyl-l-methionine (SAM, Amersham Bioscience), 50 mM Tris-HCl (pH 8.0), 20 mM KCl, 5 mM DTT, and 4 mM EDTA. The reaction mixture was incubated for 1.5 h at 37 °C and then heated with SDS loading buffer and analyzed using SDS-PAGE electrophoresis and immunoblotting.

### Measurement of FAO activity

FAO activity in HK-2 cells was measured using FAO blue reagent (FDV-0033, Funakoshi Co, Ltd., Tokyo, Japan) (11). The cells in a 96-well plate were incubated in HBSS containing 10 mM FAO blue for 2 h, washed twice with HBSS, and measured using a Spectramax M2 plate reader (excitation/emission = 405 nm/465 nm).

### Proximity ligation assay (PLA)

The Duolink In Situ Red Starter Kit Mouse/Rabbit was used for PLA, in accordance with the manufacturer’s instructions. Briefly, cells were seeded onto 24-well chamber slides, rinsed with ice-cold PBS and methanol at −20 °C for 10 min, and then permeabilized with 0.1% Triton X-100. Primary antibodies were added, and the cells were incubated for 2 h at 37 °C after washing and blocking for 30 min. The mixture was incubated at 37 °C for 1 h after adding PLA samples. Following two rounds of washing, the cells were amplified using PCR and incubated for 30 min at 37 °C with ligation reagent. The nuclei were stained with DAPI, and an Olympus FV3000 laser confocal microscope was used to obtain images. The ImageJ program was used to analyze the images (National Institutes of Health).

### WB analysis

Standard immunoblotting protocols were performed as previously described [29]. Protein lysates from cellular or tissue samples were prepared using cold RIPA lysis buffer. Protein concentrations were measured using Bradford reagent (Bio-Rad Laboratories). Proteins were then separated via SDS-PAGE and transferred to PVDF membranes, which were blocked with 5% skim milk for 1 h at 25 °C before incubation with primary antibodies for 12 h at 4 °C. After washing with TBST, the membranes were treated with secondary antibody (Proteintech) for 2 h at room temperature. Detection was performed using an enhanced chemiluminescent reagent (Millipore). Semi-quantitative analysis of protein expression was conducted using ImageJ software, with GAPDH as a loading control.

### Quantitative real-time polymerase chain reaction (qRT-PCR) analysis

Cellular RNA was extracted from mouse kidney tissues and HK-2 cells. Both tissue and cellular RNA were extracted using the TRIzol reagent (15596026CN, Invitrogen) following the manufacturer’s protocol. cDNA was synthesized using the Transcription First-Strand cDNA Synthesis Kit (Roche Diagnostics, Basel, Switzerland; 04896866001). cDNA fragments were amplified with a SYBR Green PCR kit (Roche, 04707516001) on a Roche LightCycler®480 detection system. The primers used in this study are listed in Table S3. GAPDH was used as the endogenous reference gene.

### Histopathology

Kidneys were harvested and fixed with 4% paraformaldehyde overnight to dehydrate the wax and transected into 5.0 μm thick sections using a sliding microtome (Leica, Nussloch, Germany). The sections were stained with hematoxylin and eosin after the paraffin was removed with xylene to assess the degree of kidney injury. Tubular injury was scored on a scale of 0–4: 0 = none, 1 = 0–25%, 2 = 25–50%, 3 = 50–75%, and 4 = > 75% of affected renal tubules. CaOx crystal deposition in the kidneys was detected using von Kossa staining. ImageJ software (ImageJ 1.53s, National Institutes of Health, Bethesda, MD, USA) was used to quantify the percentage of CaOx crystal deposition area in each kidney. The apoptosis level was detected using Click-iT™ TUNEL Alexa Fluor Imaging Assays Kit (C10246, Invitrogen).

Sections were incubated with primary antibodies in PBS overnight at 4 °C for immunohistochemistry. These were assessed with an HRP-conjugated polymer system (K5007, Dako, Germany) and a Leica DM2000 scanner (Leica Biosystems, Wetzlar, Germany). Relative expression levels were quantified using ImageJ software (National Institutes of Health).

For immunofluorescence, primary antibodies were incubated overnight at 4 °C after treatment with TRITC-, CY3-, or CoraLite488-conjugated goat secondary antibodies (Proteintech, China). A fluorescence microscope (Nikon, Tokyo, Japan) and a Leica DM2000 scanner (Leica Biosystems, Wetzlar, Germany) were used for observation and analysis. Relative expression levels were quantified using ImageJ software (National Institutes of Health).

### Cell immunofluorescence

Immunofluorescence staining was used to confirm the co-localization of PRMT1 and UBE2m, and the expression level of PRMT1 in the TECs of different treatment groups was detected. Intracellular lipid levels were detected using BODIPY FL Dye (GC42959; GLPBIO). Briefly, cells on coverslips were fixed with 4% paraformaldehyde for 15 min at 25 °C. They were then permeabilized in 0.2% Triton X-100 for 10 min and blocked with 8% goat serum for 1 h at room temperature. Cells on coverslips were incubated with primary antibodies overnight at 4 °C and secondary antibodies for 60 min at 37 °C. The nuclei were stained with DAPI. Finally, co-localization was examined using an Olympus FV3000 laser confocal microscope. Images were quantified in a blinded manner using the ImageJ software (National Institutes of Health).

### Transmission electron microscopy (TEM)

Fresh renal tissues measuring 1 mm³ were fixed for 2 h in 2.5% glutaraldehyde in a 0.05 M sodium cacodylate buffer at pH 7.2 and room temperature (25 °C). They were then treated for 2 h with a 0.1 M sodium cacodylate buffer containing 2% OsO_4_, followed by an overnight treatment with 1% aqueous uranyl acetate for 18 h. The specimens underwent dehydration through an ethanol series, were embedded in epoxy resin at 40 °C, and sectioned into ultrathin slices ~60–80 nm thick. These slices were double-stained with uranium lead at 4 °C for 20 min before visualization and imaging using TEM (Hitachi, Ltd.).

### Oxygen consumption rate (OCR)

The mitochondrial pressure was measured using a Seahorse XFe24 extracellular flux analyzer (Agilent Technologies). Single-cell suspension of 100 µL 10^5^/mL was incubated at 37 °C until adherent to the wall. Each cell was coated with three compound pores. According to the experimental requirements, the mixture was cultured with or without COM in a conventional incubator for 24 h and then tested. After replacing the special test medium with the hippocampal medium, the culture was incubated in a non-CO_2_ incubator for 1 h. According to the experimental design, 75 µL was added via four dosing tanks per well. The concentrations of various respiratory chain inhibitors were 1 μmol/L oligomycin, 1 μmol/L FCCP, and 1 μmol/L antimycin A. Mitochondrial stress test parameters, such as basal respiration, maximum respiration, spare capacity, ATP production, proton leakage, and non-mitochondrial respiration, were measured as the OCR.

### Statistical analysis

GraphPad Prism (9.0.0) was used for data analysis. All experimental data were calculated as mean ± SD. The appropriateness of statistical tests was ensured by examining data distribution and approximate homogeneity of variance. Student’s *t* tests were conducted to assess differences between the two groups, while ordinary one-way ANOVA was utilized to evaluate differences among multiple groups. When variance between groups was suspected to differ, we used nonparametric tests to avoid potential bias. The relationship between the two variables was examined using Pearson’s correlation and linear regression analysis. Statistical significance was set at *p* < 0.05. Statistical details, including the test used and exact *p* values, are provided in the figure or figure legends.

## Results

### PRMT1 is upregulated and regulates fatty acid metabolism in kidneys with CaOx crystal-induced injury

We analyzed single-cell RNA-sequencing data from kidney specimens of patients with stones and performed cell type identification and KEGG enrichment analysis for genes significantly and specifically expressed in each cell cluster (Fig. S1B, C) to investigate the pathophysiological mechanisms of CaOx stone-induced kidney injury. As the most commonly damaged cell type in kidney injuries, we focused on renal TECs. As shown in Fig. S1C, clusters 1, 3, and 19 represent TECs and enriched pathways of “Metabolism pathways,” “Glyoxylate and dicarboxylate metabolism,” “PPAR signaling,” and other pathways during the injury, which provided us with hints for subsequent studies. Previous studies have shown that hereditary oxalate deposition disorder, characterized by CaOx nephrolithiasis, renal failure, and oxalate crystal deposits in various tissues and organs throughout the body, is an inborn defect in glyoxylate metabolism. Therefore, we established a mouse model of CaOx crystal-induced kidney injury using intraperitoneal injection of glyoxylate, in compliance with some previous studies [30–32].

**Fig. 1:**
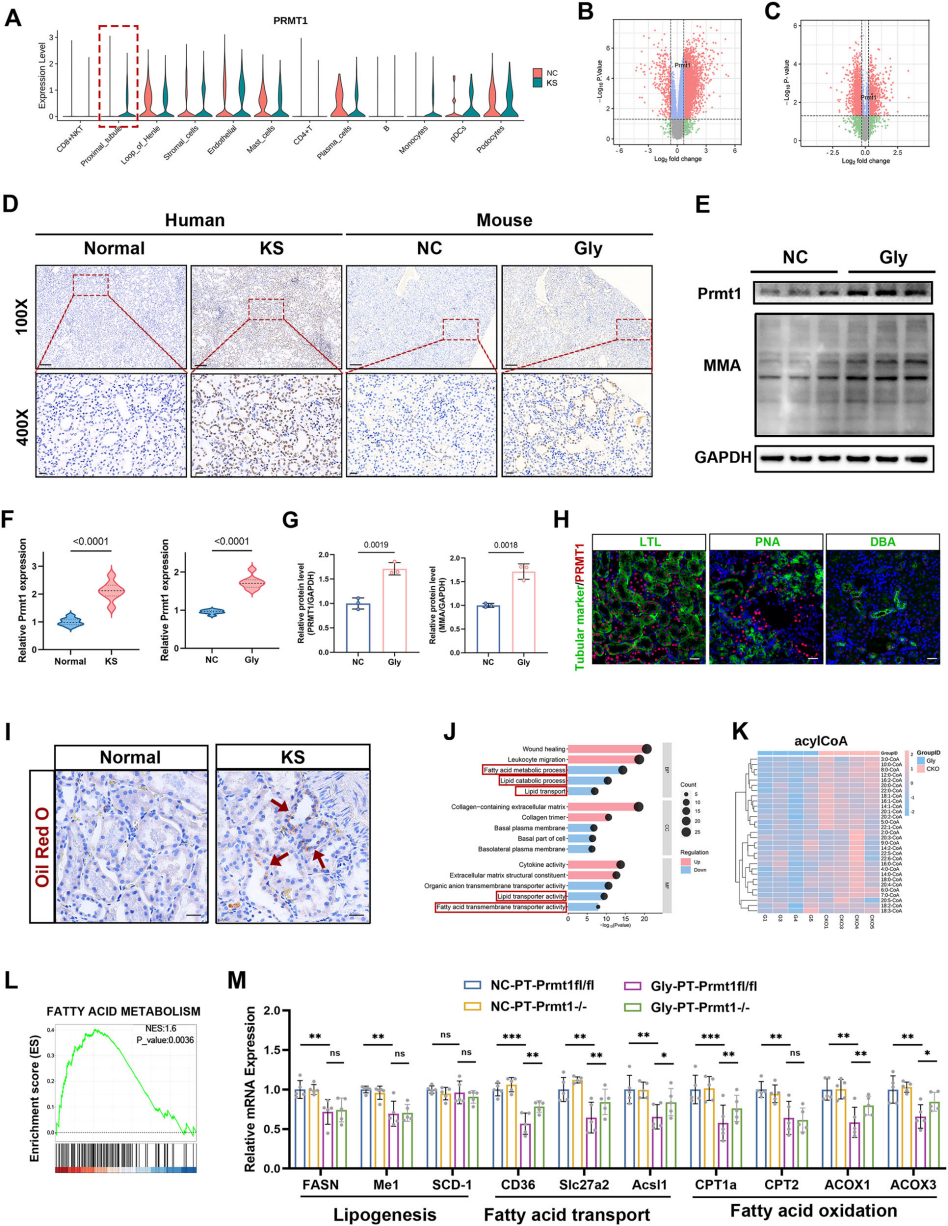
PRMT1 is upregulated and regulates fatty acid metabolism in kidneys with calcium oxalate crystal-induced injury. **A** The violin plot showing the expression level of PRMT1 in various cell clusters from the NC and the KS groups. **B**, **C** The volcano plot of different expression genes or proteins assayed using RNA- or proteome sequencing of kidney tissues. PRMT1 was significantly increased at both the RNA and protein levels. **D** Representative immunohistochemical staining images (100× and 400×) and quantitative analysis (**F**) of the expression of PRMT1 in the nonfunctioning kidney of patients and in kidneys with CaOx crystals injury of mice. Scale bars = 50 μm. **E** Representative western blot banding and quantitative analysis (**G**) of the expression levels of PRMT1 and MMA in the NC and the Gly groups. **H** Representative images of immunofluorescence co-staining of PRMT1 (red) and renal tubular markers LTL, PNA, and DBA (green) in kidneys of mice. Scale bars = 50 μm. **I** Oil Red O staining of kidney sections from the indicated groups. **J** GO enrichment analysis of RNA-sequencing data comparing the expression levels between NC and the Gly group. **K** Heat map showing differential content of acyl-CoAs between the NC and the Gly groups. **L** GSEA enrichment analysis of differential proteins between the Gly group and the CKO group, assayed by proteome sequencing of kidney tissues. **M** Relative mRNA levels of key molecules of lipogenesis, fatty acid transport, and oxidation from the indicated groups. Significance was assessed using two-way ANOVA or t-tests. Data are presented as mean ± SD.

Further analysis of single-cell RNA data revealed that PRMT1 was significantly upregulated in TEC clusters (Fig. 1A). We collected renal tissues that were used for proteomics and transcriptome sequencing. The volcano diagram results suggested that PRMT1 was significantly upregulated at both the protein and mRNA levels in the Gly group compared to that of the negative control group (Fig. 1B, C). Immunohistochemical results showed that PRMT1 was significantly upregulated in the kidneys of both patients with stones and mice with crystal kidney injury and that it was mainly localized in TECs (Fig. 1D, F), as verified using WB (Fig. 1E, G). We co-stained kidney sections with LTL, PNA, and DBA to clarify the location of PRMT1 expression in the kidney. We observed that PRMT1 was most expressed in proximal TECs and almost no expression was observed in distal TECs or collecting duct cells (Fig. 1H**)**. We established an in vitro model by treating HK-2 cells with CaOx. The immunofluorescence results showed that the level of PRMT1 was significantly increased in the nuclei of COM-treated HK-2 cells (Fig. S1J).

Renal tissues from mice with CaOx crystal kidney injury and from negative control mice were collected for untargeted metabolomics. Data analysis indicated significant differences in the metabolites between the Gly and negative control groups (Figure S1D, E), and the differences in amino acid and lipid metabolism were the most significant. “Lipid metabolism” related substances and “Lipids and lipid-like molecules” were significantly increased in the Gly group (Fig. S1F–H). The above results were also verified using oil red O staining, and lipid accumulation was observed in the kidneys of patients with stones (Fig. 1I). Transcriptome GO enrichment analysis comparing DEGs from the Gly and NC groups in mice showed that the “Fatty acid metabolic process,” “Lipid catabolic process,” “Lipid transporter activity,” “Fatty acid transmembrane transporter activity,” were enriched and downregulated significantly (Fig. 1J). Furthermore, GO and KEGG transcriptomic analyses of DEGs between the KS and NC groups in human similarly demonstrated significant suppression of key lipid-related pathways as shown in Fig. S1I.

We generated renal TEC-specific PRMT1 KO mice (PT-Prmt1−/−, CKO) to investigate the role of PRMT1 in lipid metabolism regulation and conducted proteomic sequencing on samples from the Gly and “Gly-CKO” groups. Acyl-CoA is a product of the fatty acid metabolism. We measured the global acyl-CoA content in the Gly and CKO groups and showed that PRMT1 knockdown in TECs increased acyl-CoA content, indicating increased fatty acid metabolism (Fig. 1K). GSEA analysis indicated that fatty acid metabolism was significantly upregulated in the CKO group compared to that in the Gly group (Fig. 1L). The mRNA levels of key molecules involved in lipid synthesis, fatty acid transport, and FAO were measured to explore which lipid metabolism processes are regulated. RT-PCR analysis revealed that fatty acid transport and oxidation were significantly downregulated in the Gly group and rescued in the CKO group. However, no significant differences were observed in the mRNA levels of lipid synthesis-related molecules between the CKO and Gly groups, indicating that PRMT1 may be involved in regulating fatty acid transport and oxidation (Fig. 1M). The above findings suggested that PRMT1 and fatty acid metabolism might have essential roles in CaOx crystal-induced kidney injury, and that PRMT1 was involved in regulating fatty acid metabolism.

### Knockout or overexpression of PRMT1 in TECs can alleviate or aggravate CaOx-induced renal injury by regulating fatty acid metabolism

TEC-specific PRMT1 overexpression mice were generated by injecting rAAV9 carrying either a TEC-specific Ksp promoter-driven PRMT1 construct (rAAV9-Ksp-PRMT1) or an empty vector (rAAV9-Ksp-null) into the bilateral kidneys using a microliter syringe to further evaluate the role of PRMT1. A CaOx crystal-induced kidney injury model was established 3 weeks later (Fig. S2A, B). WB results and green EGFP fluorescence in kidney sections confirmed successful rAAV9-PRMT1 transduction (Fig. S2C–E). Simultaneously, TEC-specific PRMT1 KO mice were used to construct a CaOx crystal-induced kidney-injury mouse model, and the KO efficiency was detected using WB and DNA agarose gel electrophoresis (Fig. S2G–J). We knocked down PRMT1 in HK-2 cells using a lentiviral vector and overexpressed PRMT1 in vitro using an adenoviral (ADV) vector carrying PRMT1 DNA; the transfection efficiency was verified using PCR and WB (Fig. S3A–C).

**Fig. 2:**
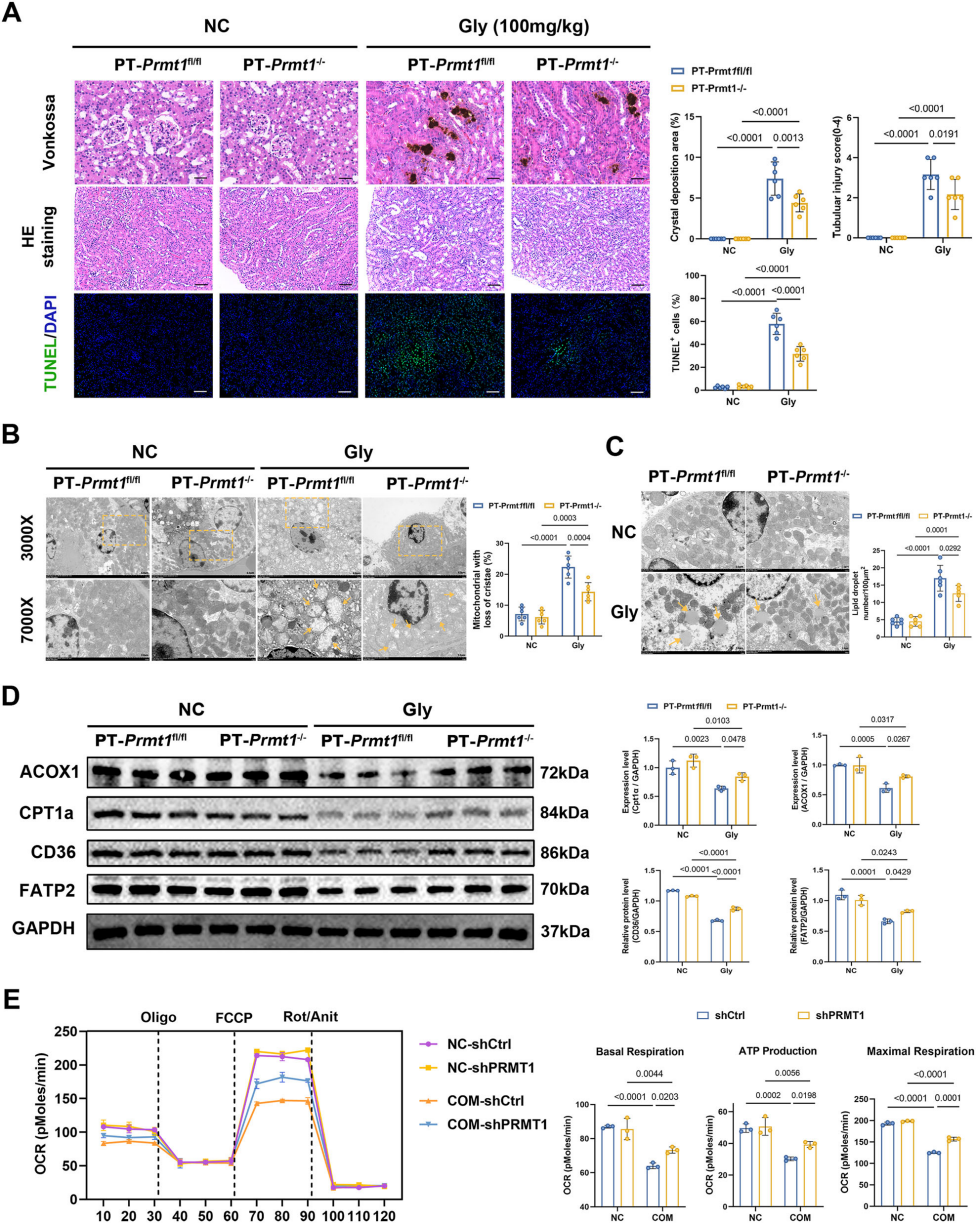
Knockout of PRMT1 in tubular epithelial cells can alleviate calcium oxalate-induced renal injury by improving fatty acid metabolism. **A** Representative histology images of the indicated groups: Vonkossa staining indicates the area and location of calcium oxalate crystals deposition (400×); HE staining shows the degree of renal tubular injury (200×); TUNEL staining shows the relative number of apoptotic renal cells (200×). Scale bars = 50 μm. **B**, **C** Representative TEM images demonstrate the degree of mitochondrial damage and lipid droplet accumulation. Scale bars = 500 nm. **D** Representative western blot banding and quantitative analysis of the expression levels of ACOX1, CPT1a, CD36, and FATP2. **E** Mitochondrial oxidative capacity was measured in real time after knockdown of PRMT1 or COM treatment in HK-2 cells; Quantification of basal respiration, ATP production-coupled respiration, and maximal respiration. Significance was assessed using two-way ANOVA tests. Data are shown as mean ± SD.

**Fig. 3:**
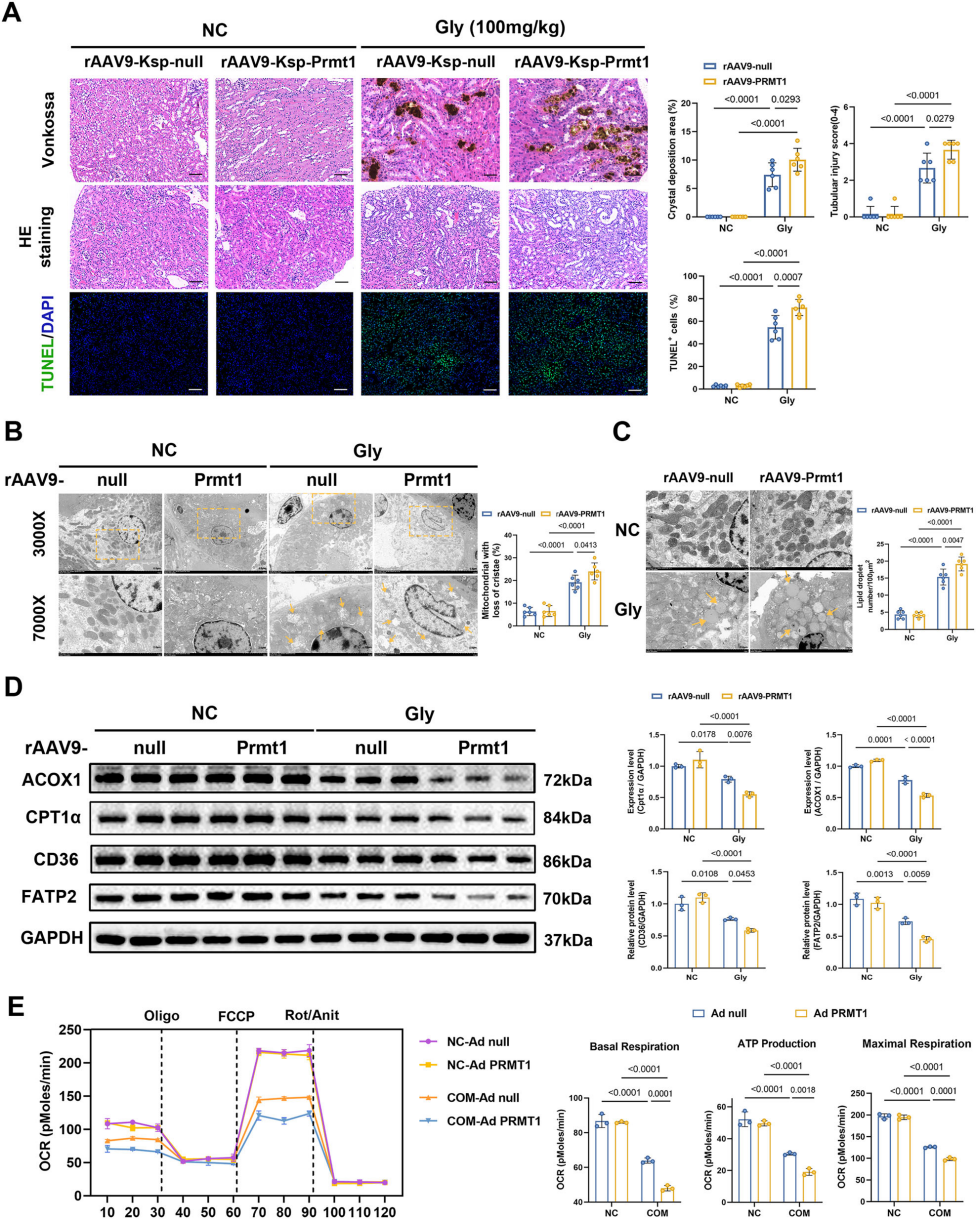
Overexpression of PRMT1 in tubular epithelial cells can aggravate calcium oxalate-induced renal injury by inhibiting fatty acid metabolism. **A** Representative histology images of the indicated groups: Vonkossa staining indicates the area and location of calcium oxalate crystal deposition (1X and 200X); HE staining shows the degree of renal tubular injury (200X); TUNEL staining shows the relative number of apoptotic renal cells (200X). Scale bars = 50 μm. **B**, **C** Representative TEM images demonstrate the degree of mitochondrial damage and lipid droplet accumulation. Scale bars = 500 nm. **D** Representative western blot bandings show the expression levels of ACOX1 and CPT1α. **E** Mitochondrial oxidative capacity was measured in real time after overexpression of PRMT1 or COM treatment in HK-2 cells; basal respiration, ATP production-coupled respiration, and maximal respiration were quantified. Significance was assessed using two-way ANOVA tests. Data are shown as mean ± SD.

We observed CaOx crystal deposition, tubular dilatation, inflammatory cell infiltration, and apoptosis in the Gly group. These injuries were alleviated by PRMT1 knockdown (Fig. 2A), whereas PRMT1 overexpression aggravated crystal deposition and damage (Fig. 3A). TEM revealed mitochondrial cristae disruption, swelling, rupture, and intracellular lipid droplet accumulation, suggesting impaired lipid metabolism and energy production in TECs. These effects were aggravated by PRMT1 overexpression but attenuated by PRMT1 knockdown (Fig. 2B, C, Fig. 3B, C). We then detected key fatty acid transport molecules (CD36 and FATP2) and key FAO molecules (CPT1a and ACOX1). Consistent with the mRNA levels, the WB results showed that their expressions were significantly downregulated in the Gly group, recovered after PRMT1 knockdown, and further decreased after PRMT1 overexpression (Fig. 2D, Fig. 3D). In vitro, CD36, FATP, CPT1a, and ACOX1 were significantly downregulated in HK-2 cells treated with COM, recovered after PRMT1 knockdown, and further decreased after PRMT1 overexpression (Fig. S6D, E). The mitochondrial OCR was assessed using a Seahorse analyzer. OCR was significantly blocked, and ATP production, basal respiration, and maximal respiration were significantly decreased after COM treatment compared to that in the control group. These effects were restored by PRMT1 knockdown and further worsened by PRMT1 overexpression (Fig. 2E, Fig. 3E). As expected, BODIPY staining confirmed that COM treatment increased lipid accumulation, which was rescued by PRMT1 knockdown but further induced by PRMT1 overexpression (Fig. S3A, B). These findings suggested that PRMT1 in TECs may be involved in the CaOx crystals-induced renal injury by regulating fatty acid metabolism.

Furthermore, to explore the potential therapeutic relevance of modulating lipid metabolism, we administered atorvastatin, a commonly used lipid-lowering drug, to mice with CaOx-induced renal injury. Atorvastatin treatment significantly alleviated renal injury, as evidenced by decreased KIM-1 expression levels and reduced histological damage (Fig. S4C, D). Additionally, atorvastatin partially restored the expression of key enzymes involved in FAO and reduced lipid accumulation (Fig. S4E, F). These findings support the notion that targeting lipid metabolic dysfunction may offer a feasible strategy to mitigate CaOx crystal-induced kidney damage.

### PRMT1 interacts with and methylates UBE2m and this interaction is enhanced in TECs with COM treatment

**Fig. 4:**
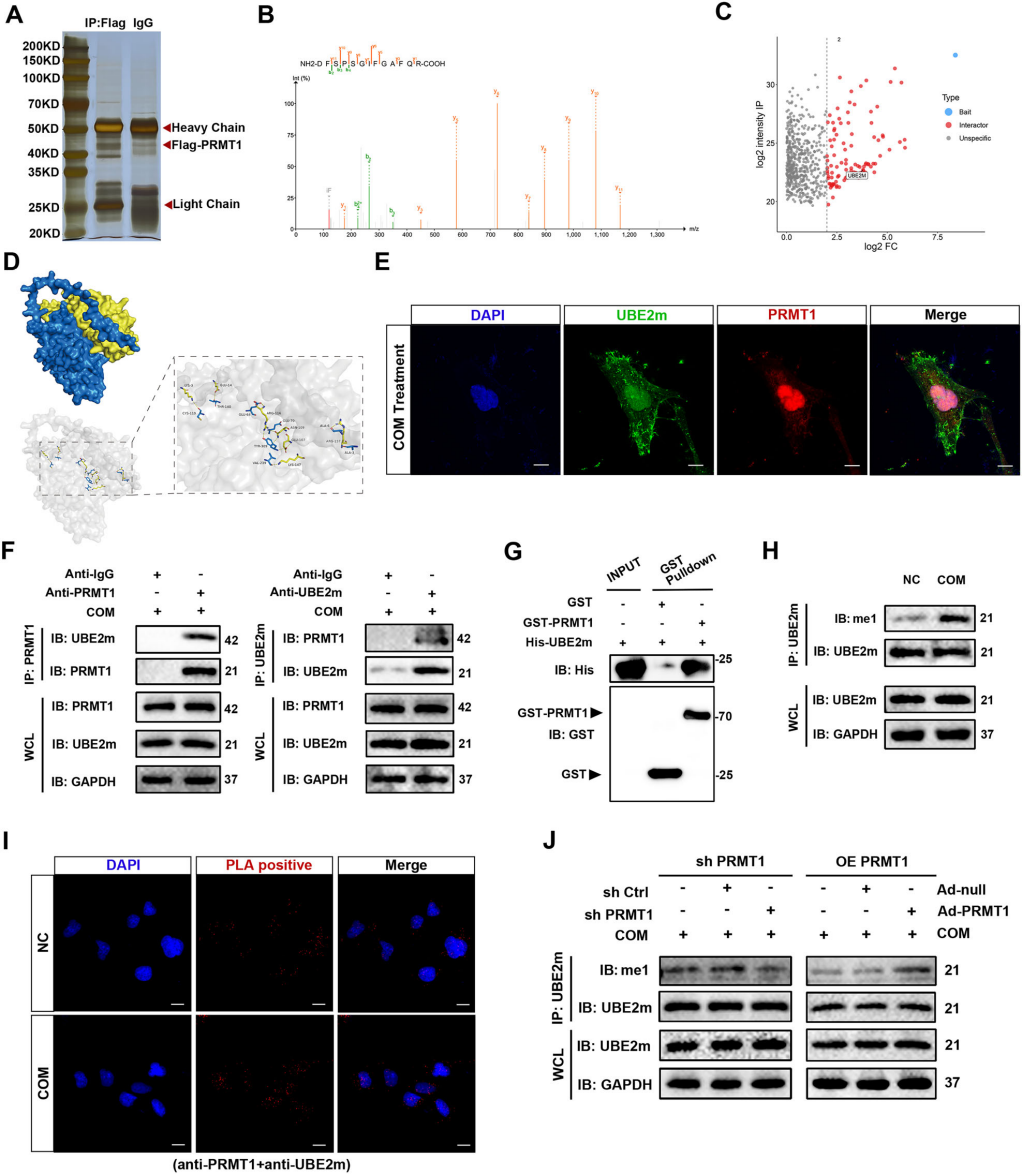
PRMT1 interacts with and methylates UBE2m, and this interaction is enhanced in tubular epithelial cells with COM treatment. **A** Silver-stained SDS-PAGE gel of the immunoprecipitation product. **B** MS/MS spectra of the peptide “FSPSGIFGAFQR-COOH.” Peaks in color are the detected b (green) and y (red) ions. **C** The volcano plot indicating potential target proteins of PRMT1. **D** Molecular docking pattern diagram of PRMT1 and UBE2m. **E** Representative images of immunofluorescence staining of UBE2m (green) and PRMT1 (red) in HK-2 cells treated with COM. Scale bars = 50 μm. **F** Reciprocal co-IP analysis of PRMT1 and UBE2m in HK-2 cells with COM treatment. IgG was used as a negative control. **G** Recombinant GST-PRMT1 was incubated with recombinant His-UBE2m, followed by GST-pulldown and immunoblotting analysis with GST and His antibodies. **H** Endogenous UBE2m was immunoprecipitated in cells from the indicated groups. The mono-methylation (me1) level of UBE2m was determined using immunoblotting. **I** PLA detection of PRMT1 and UBE2m interaction in HK-2 cells from the indicated groups. Scale bars = 50 μm. **J** The me1 level of UBE2m was determined using immunoblotting after UBE2m was immunoprecipitated in HK-2 cells with PRMT1 knockdown or overexpression.

We sought to identify potential binding proteins of PRMT1 to elucidate the mechanism of PRMT1 in regulating fatty acid metabolism. HK-2 cells were transfected with a FLAG-tagged PRMT1 plasmid (Fig. S5F), followed by immunoprecipitation of whole-cell lysates using a FLAG primary antibody. The immunoprecipitation efficiency was verified using WB (Fig. S5G), and protein bands were visualized using silver staining (Fig. 4A). The immunoprecipitated complexes were then subjected to LC-MS analysis, leading to the identification of 99 interacting proteins with a fold change ≥4. The results of GO and KEGG enrichment analyses showed that “Ubiquitin-like modifier activating enzyme activity” and “Ubiquitin-mediated proteolysis” were significantly enriched (Fig. S5H). This drew our attention to the ubiquitin-related protein UBE2m, which is a ubiquitin-conjugating enzyme essential for the neddylation that was significantly enriched (Fig. 4B, C). We performed molecular docking analysis and bidirectional co-immunoprecipitation to verify whether PRMT1 and UBE2m interact. Molecular docking successfully predicted a stable PRMT1–UBE2m interaction (Fig. 4D). In addition, PRMT1 and UBE2m showed co-localization in HK-2 cells (Fig. 4E). Both PRMT1 and UBE2m elicited strong immune responses in COM-stimulated HK-2 cells. These response proteins were detected using WB. PRMT1 co-precipitated UBE2m in HK-2 cells but not in control cells with IgG antibody. Reverse co-immunoprecipitation confirmed that UBE2m precipitated PRMT1 in HK-2 cells (Fig. 4F). We performed GST pull-down experiments by co-incubating recombinant human GST-PRMT1 protein and His-UBE2m protein to determine whether PRMT1 binds UBE2m directly. Notably, a direct interaction between PRMT1 and UBE2m was observed (Fig. 4G). Furthermore, we found that the expression of UBE2m was PRMT1-independent (Fig. S5I), so we investigated whether PRMT1 could methylate UBE2m. Immunoprecipitation assays revealed elevated UBE2m methylation than that in the NC group (Fig. 4H). As expected, the methylation level of UBE2m significantly decreased after PRMT1 knockdown and significantly increased after PRMT1 overexpression (Fig. 4J). PLA revealed an enhanced interaction between PRMT1 and UBE2m in COM-treated HK-2 cells compared with that in the control cells (Fig. 4I). Collectively, these results demonstrated that PRMT1 interacted with and methylated UBE2m. This interaction was enhanced in TECs treated with COM.

### PRMT1 regulates fatty acid metabolism by methylating UBE2m at R169 in TECs

**Fig. 5:**
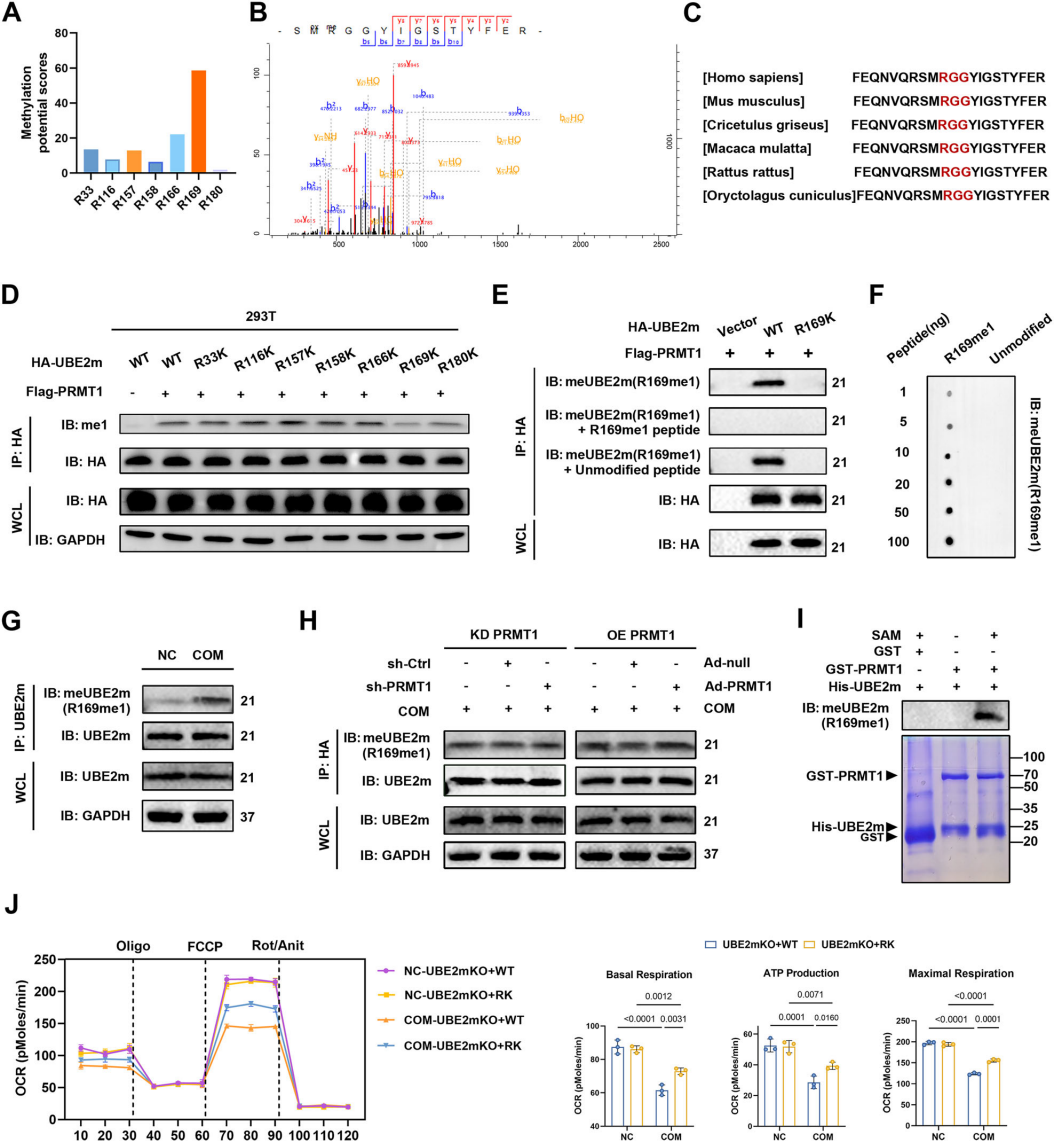
PRMT1 regulates fatty acid metabolism by methylating UBE2m at R169 in tubular epithelial cells. **A** The scores of potential methylation sites of UBE2m predicted using the GPS-MSP tool. **B** MS identification of R169 mono-methylation of HA-UBE2m immunopurified by HA beads. **C** Sequence alignment of UBE2m protein from indicated species. **D** HA-UBE2m WT or different point mutants were co-transfected with FLAG-PRMT in HEK293T cells. The me1 level of UBE2m was measured by immunoblotting following immunopurification. **E** The me1 of immunopurified HA-UBE2m was measured using a site-specific antibody against R169 mono-methylation (meUBE2m (R169me1)). Antibody efficacy and specificity were examined by pre-incubating with the R169me1 peptide or the unmodified peptide prior to application. **F** Dot blot analysis of different amounts of R169me1 peptide or unmodified peptide by a site-specific antibody against meUBE2m (R169me1). **G** Endogenous UBE2m was immunoprecipitated in cells from the indicated groups. The R169me1 level of UBE2m was determined using immunoblotting. **H** The R169me1 level of UBE2m was determined using immunoblotting after UBE2m was immunoprecipitated in HK-2 cells with PRMT1 knockdown or overexpression. **I** Recombinant GST-PRMT1 and His-UBE2m proteins were incubated with or without SAM to detect the methylation of UBE2m mediated by PRMT1 in vitro. The reaction mixture was then subjected to CBB staining and immunoblotting with meUBE2m (R169me1) antibody. **J** Mitochondrial oxidative capacity was measured in real time after reintroduced UBE2m WT or RK treatment in UBE2m KO HK-2 cells; basal respiration, ATP production-coupled respiration, and maximal respiration were quantified. Significance was assessed using two-way ANOVA tests. Data are shown as mean ± SD.

Next, we predicted the position of UBE2m arginine methylation by using the GPS-MSP tool [33], among which R169 had the highest methylation score (Fig. 5A). MS analysis of immunoprecipitated UBE2m validated mono-methylation of UBE2m at R169 (Fig. 5B). In addition, R169 and its adjacent amino acids have significant conservation across various species (Fig. 5C) and closely match the common RGG/RG methylation motif of PRMT1. We generated UBE2m R169K mutants and compared their methylation levels with that of the wild-type UBE2m using immunoprecipitation to validate this finding. The R169K mutation significantly reduced the methylation level of UBE2m (Fig. 5D), demonstrating that R169 is the primary methylation site in TECs.

Site-specific antibodies against monomethylated R169 (meUBE2m (R169me1)) were generated, and peptide competition and dot blot analyses were used to verify the specificity to better understand the R169 methylation of UBE2m (Fig. 5E, F). This antibody specifically recognizes the mono-methylation of R169 on both exogenous and endogenous UBE2m, which was elevated in COM-treated HK-2 cells compared to that in the NC group and responded to PRMT1 manipulation (Fig. 5G, H). We conducted an in vitro methylation assay utilizing recombinant human GST-PRMT1 protein and His-UBE2m protein to ascertain if PRMT1 could directly methylate UBE2m. Methylation of His-UBE2m at R169 was observed in the presence of both GST-PRMT1 and SAM (Fig. 5I).

We knocked out UBE2m (UBE2m KO) using CRISPR/Cas9 technique, verified the knockdown efficiency using WB (Fig. S6A), and re-expressed UBE2m WT or R169K (RK) to explore whether UBE2m R169 methylation is involved in regulating fatty acid metabolism in TECs. The OCR results showed that the R169K mutation did not affect the respiration of TECs in the NC group, and the reintroduction of UBE2m WT HK-2 cells significantly blocked OCR after COM treatment. Decreased basal and maximal respiration were markedly rescued by reintroducing UBE2m WT (Fig. 5J). These results suggested that PRMT1 regulated fatty acid metabolism by methylating UBE2m at R169.

### PRMT1 regulates NEDD4-mediated PPARγ ubiquitination by methylating UBE2m in TECs

**Fig. 6:**
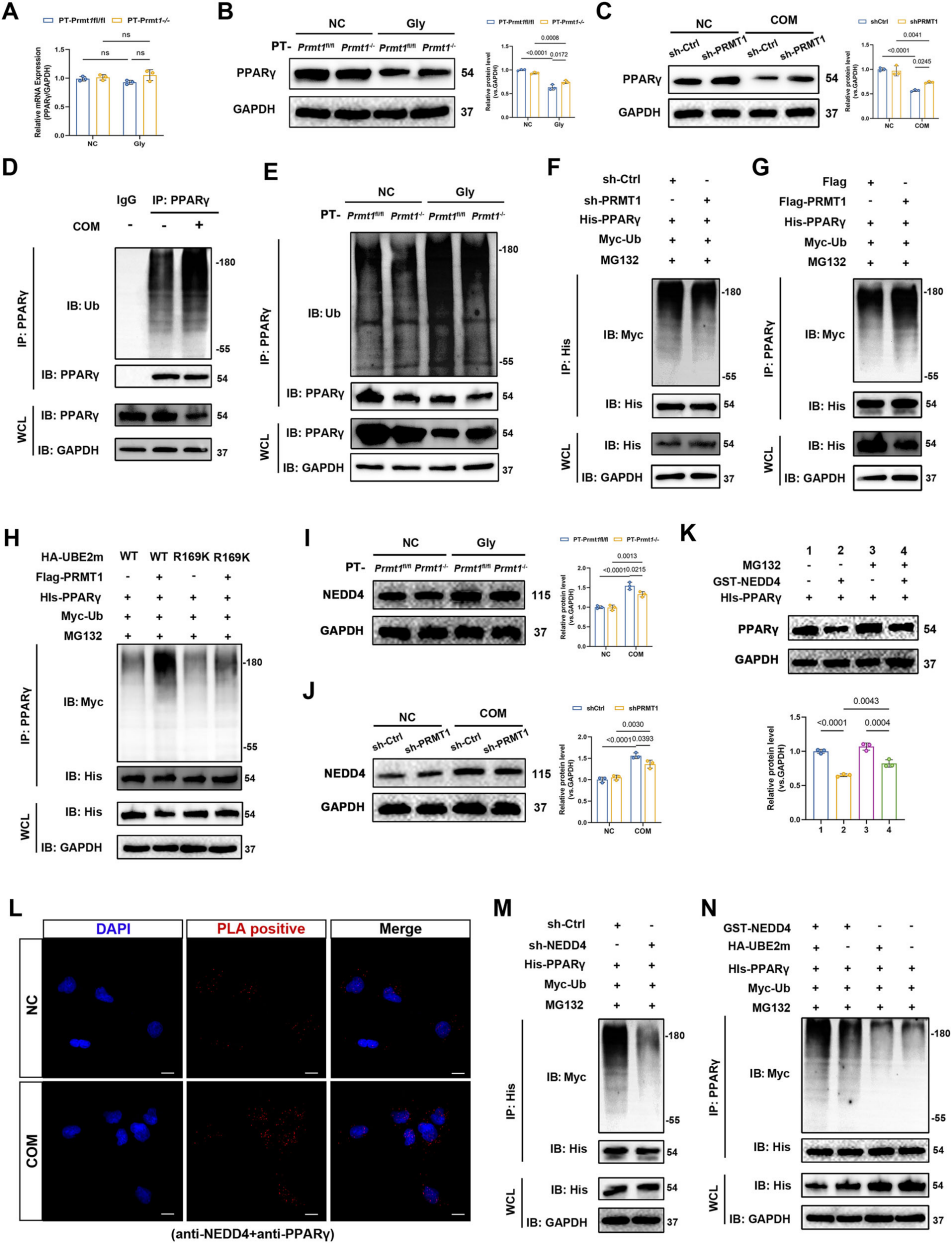
PRMT1 regulates NEDD4-mediated PPARγ ubiquitination by methylating UBE2m in tubular epithelial cells. **A** RT-PCR was used to detect the RNA levels of PPARγ from the indicated groups. Representative western blot banding and quantitative analysis show the expression levels of PPARγ from the indicated groups in mice (**B**) or in HK-2 cells (**C**). **D** Detection of endogenous ubiquitination levels of PPARγ in HK-2 cells with COM-treatment. **E** Detection of endogenous ubiquitination levels of PPARγ in CaOx crystals kidney injury in mice. **F**, **G** Detection of exogenous ubiquitination levels of PPARγ in HEK193T cells with PRMT1 knockdown or overexpression. **H** Detection of exogenous ubiquitination levels of PPARγ in HEK193T cells from the indicated groups. Representative western blot banding and quantitative analysis of the expression levels of NEDD4 from the indicated groups in mice (**I**) or HK-2 cells (**J**). **K** HK-2 cells were treated with MG132. Representative western blot banding and quantitative analysis showed the expression levels of PPARγ from the indicated groups. **L** PLA detection of NEDD4 and PPARγ interaction in HK-2 cells from the indicated groups. Scale bars = 50 μm. **M** Detection of exogenous ubiquitination levels of PPARγ in HEK193T cells with knockdown of NEDD4. **N** Detection of exogenous ubiquitination levels of PPARγ in HEK193T cells from the indicated groups. Significance was assessed using two-way ANOVA tests. Data are shown as mean ± SD.

Additionally, we explored how the PRMT1/UBE2m axis inhibits fatty acid metabolism. Analysis of sequencing data and previous experiments have confirmed that molecules related to fatty acid metabolism are downregulated at both mRNA and protein levels. The PPAR family is the key transcription factor involved in lipid metabolism, and scRNA-seq also indicated that “PPAR signaling” was significantly enriched and specifically expressed in TEC clusters. We found that PPARγ was regulated by PRMT1 both in vitro and in vivo (Fig. 6A–C). However, its methylation level was not regulated by PRMT1 (Fig. S6B), nor was the neddylation level altered (Fig. S6C). We then investigated whether PPARγ was ubiquitinated and observed that the level of PPARγ ubiquitination was significantly increased in COM-treated HK-2 cells compared with that of the control group (Fig. 6D). Similarly, PPARγ ubiquitination was significantly increased in mice with CaOx crystal kidney injury. This effect was suppressed after PRMT1 knockdown (Fig. 6E). Moreover, our findings indicated that either overexpression or knockdown of PRMT1 in HK-2 cells could sufficiently enhance or suppress the ubiquitination of PPARγ (Fig. 6F, G). Subsequently, we explored whether UBE2m methylation was involved in regulating PPARγ ubiquitination. By reintroducing UBE2m WT or RK in UBE2m KO, we found that PPARγ ubiquitination was inhibited after blocking UBE2mR169 methylation (Fig. 6H). Overexpression of UBE2m in HK-2 cells did not increase the neddylation level of PPARγ (Fig. S6C), indicating that PRMT1 is involved in regulating PPARγ ubiquitination by methylating UBE2m.

UbiBrowser 2.0 database indicated that NEDD4, VHL, SMURF1, and SIAH2. are the classical E3 ubiquitin ligases of PPARγ (Fig. S6D) and promote the degradation of PPARγ through the ubiquitin-proteasome pathway [34]. We examined several E3 ubiquitin ligases of PPARγ and found that only NEDD4 was upregulated in our model (Fig. S6E). We further explored the regulation of NEDD4 expression and found that PRMT1 knockdown inhibited the expression of NEDD4 (Fig. 6I, J) without influencing its transcription (Fig. S6F) or methylation (Fig. S6G) levels. The PLA results showed that NEDD4 and PPARγ interacted in HK-2 cells, which was enhanced in COM-treated HK-2 cells (Fig. 6L). We further validated the ubiquitination of PPARγ by NEDD4. We observed a rapid degradation of PPARγ protein in the NEDD4 overexpression group, which was ameliorated upon treatment of HK-2 cells with the proteasome inhibitor MG132 (Fig. 6K). Moreover, NEDD4 knockdown in HK-2 cells was adequate to inhibit the ubiquitination of PPARγ (Fig. 6M). Subsequently, we explored whether UBE2m regulated PPARγ ubiquitination through NEDD4. We found that UBE2m failed to affect PPARγ ubiquitination inhibition in the absence of NEDD4 (Fig. 6N). These results suggested that PRMT1 regulated NEDD4-mediated ubiquitination of PPARγ by methylating UBE2m.

### Methylated UBE2m inhibits PPARγ-mediated fatty acid metabolism by neddylating NEDD4 in TECs

**Fig. 7:**
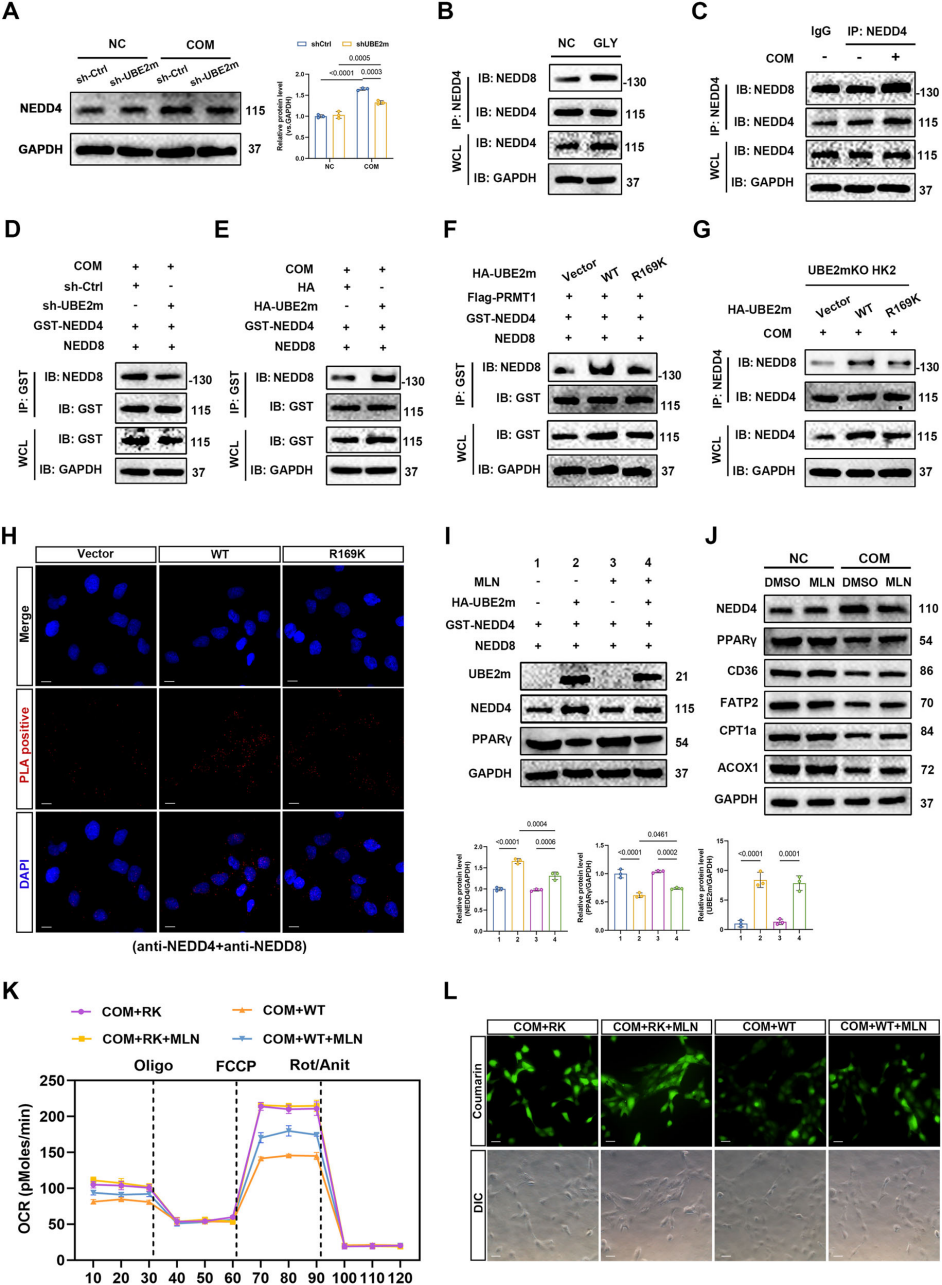
Methylated UBE2m inhibits PPARγ-mediated fatty acid metabolism by neddylating NEDD4 in tubular epithelial cells. **A** Representative western blot banding and quantitative analysis of the expression levels of NEDD4 from the indicated groups in HK-2 cells. **B** Detection of endogenous neddylation levels of NEDD4 from the indicated groups in mice or in HK-2 cells (**C**). **D**, **E** Detection of exogenous neddylation levels of NEDD4 in HEK193T cells with UBE2m knockdown or overexpression. **F** Detection of exogenous neddylation levels of NEDD4 in HEK193T cells transfecting UBE2m WT or RK. **G** Detection of endogenous neddylation levels of NEDD4 in UBE2mKO HK-2 cells re-introducing UBE2m WT or RK. **H** PLA detection of NEDD4 and NEDD8 interaction in HK-2 cells from the indicated groups. Scale bars = 50 μm. **I** HK-2 cells were treated with MLN4924. Representative western blot banding and quantitative analysis of the expression levels of UBE2m, NEDD4, and PPARγ from the indicated groups in HK-2 cells; and the expression levels of NEDD4, PPARγ, CD36, FATP2, CPT1α, and ACOX1 from the indicated groups in HK-2 cells (**J**). **K** Mitochondrial oxidative capacity was measured in real time after reintroduced UBE2m WT or RK treatment in UBE2m KO HK-2 cells, with or without MLN. **L** FAO blue staining demonstrates the degree of FAO in HK-2 cells from the indicated groups. Significance was assessed using two-way ANOVA tests. Data are shown as mean ± SD.

We further investigated how UBE2m, a key ubiquitin-conjugating enzyme essential for neddylation, regulates NEDD4-mediated PPARγ ubiquitination. UBE2m neddylates classical substrates (e.g., cullin family members) and non-cullin substrates, including p73 and cGAS [35, 36]. Therefore, we investigated whether NEDD4 was neddylated by UBE2m. WB results demonstrated that the increased expression of NEDD4 after COM treatment was restored after the knockdown of UBE2m in HK-2 cells (Fig. 7A). The neddylation level of NEDD4 increased both in vitro and in vivo (Fig. 7B, C). Furthermore, we found that the neddylation level of NEDD4 decreased after UBE2m knockdown and increased after UBE2m overexpression. This indicated that the neddylation level of NEDD4 was regulated by UBE2m (Fig. 7D, E). We further tested whether the methylation of UBE2m affects NEDD4 neddylation using exogenous and endogenous assays. Compared to the UBE2m RK, the re-introduction of UBE2m WT promoted NEDD4 neddylation in UBE2mKO HK-2 cells, while NEDD4 neddylation could not be induced by the vector alone (Fig. 7F, G). The PLA results suggested that the reintroduction of UBE2m WT promoted NEDD4 and NEDD8 binding in UBE2mKO HK-2 cells. The binding of NEDD4 and NEDD8 was not observed when only the vector was introduced (Fig. 7H). These results suggested that methylation of R169 in UBE2m promoted the neddylation of NEDD4.

Pevonedistat (MLN4924) is a highly potent and selective inhibitor of neddylation (IC50 value of 4.7 nM), which can block neddylation modification. When HK-2 cells were treated with MLN4924, the UBE2m overexpression-induced increase in neddylation and expression of NEDD4 was rescued, as was the decrease in PPARγ expression induced by NEDD4 elevation (Fig. 7I). Moreover, pevonedistat treatment rescued the expression of key fatty acid transport and oxidation molecules (Figs. 7J and S7A). Compared to UBE2m RK, fatty acid metabolism and mitochondrial respiration were inhibited after re-expression of UBE2m WT, and the suppressed OCR was rescued significantly after treatment with MLN4924 (Figs. 7K and S7B). FAO blue staining also revealed that MLN4924 inhibited FAO in TECs, as quantified by fluorescence intensity (Figs. 7L and S7C). The above results demonstrated that methylated UBE2m inhibited PPARγ-mediated fatty acid metabolism by neddylating NEDD4.

### PRMT1 and methylated UBE2m are upregulated in the kidneys of patients with stones and correlate with renal injury

**Fig. 8:**
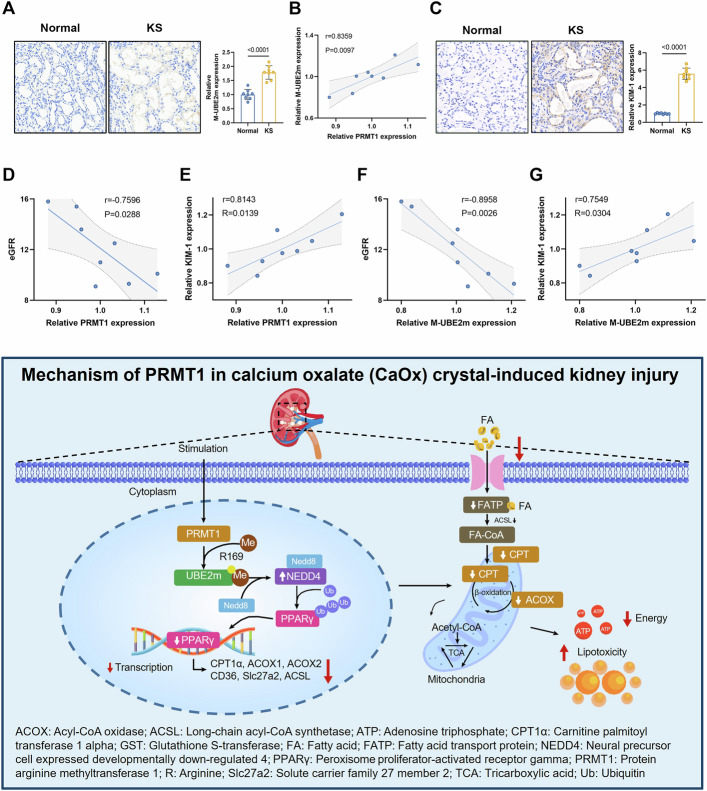
PRMT1 and methylated UBE2m are upregulated in the kidneys of patients with stones and positively correlated with renal injury markers. **A** Representative immunohistochemical staining images (400X) and quantitative analysis of the expression of M-UBE2m in the nonfunctioning kidney of patients with stones or the control. Scale bars = 50 μm. **B** Pearson correlation analysis of the expression of PRMT1 and M-UBE2m. **C** Representative immunohistochemical staining images (400X) and quantitative analysis of the expression of KIM-1 in the nonfunctioning kidney of patients with stones or the control. Scale bars = 50 μm. **D**–**G** Pearson correlation analysis of the expression of PRMT1 and eGFR (**D**), PRMT1 and KIM-1 (**E**), M-UBE2m and eGFR (**F**), M-UBE2m and KIM-1 (**G**). Significance was assessed using t-tests. Data are presented as mean ± SD.

We further evaluated the roles of PRMT1 and methylated UBE2m in kidney tissue from patients with stones. Immunostaining revealed that PRMT1 and methylated UBE2m were predominantly expressed in the renal tubular cells of patients but rarely detected in controls (Figs. 1D and 8A). Furthermore, the expression levels of PRMT1 and methylated UBE2m showed a significant positive correlation (*r* = 0.8359) (Fig. 8B). The expression of kidney injury marker KIM-1 was semi-quantified using immunohistochemistry (Fig. 8C). Further, we found that PRMT1 and methylated UBE2m were positively correlated with kidney injury marker KIM-1 (*r* = 0.8143; *r* = 0.7549) and negatively correlated with estimate glomerular filtration rate (eGFR) (*r* = −0.7596; *r* = −0.8958) (Fig. 8D–G). These results suggested that PRMT1 and methylated UBE2m are closely related to renal injury induced by stones.

## Discussion

KSs are a common urological disorder characterized by chronic inflammation and tissue injury, which significantly increases the risk of recurrence and perpetuates a vicious cycle [5]. While some therapeutic approaches have shown promise in limiting renal injury and fibrosis in clinical trials [2, 6], the overall clinical efficacy remains limited. Our study elucidated a novel PRMT1/UBE2m/NEDD4/PPARγ signaling axis that drives renal lipid metabolic dysfunction in CaOx crystal-induced kidney injury. Through integrated multi-omics approaches and rigorous mechanistic studies, we demonstrated how PRMT1-mediated post-translational modifications orchestrate a cascade of molecular events leading to TEC injury. Three key findings emerged from this study: (1) PRMT1 upregulation in injured TECs drives UBE2m methylation at R169, (2) methylated UBE2m promotes NEDD4 neddylation and subsequent PPARγ ubiquitination, and (3) this pathway is clinically relevant in stone patients, correlating with renal function decline.

Reprogramming of lipid metabolism may represent a key mechanism of kidney stone formation [37, 38]. CaOx crystal deposition impairs fatty acid metabolism in TECs, leading to insufficient ATP production, apoptosis, and necrosis. Concurrently, unmetabolized fatty acids accumulate intracellularly, contributing to lipid overload and lipotoxicity [39]. In addition, fatty acid uptake is impaired, promoting interstitial lipid accumulation and chronic inflammation, exacerbating renal injury. Qi Zhu et al. showed that PRMT1 promoted fat formation in 3T3-L1 and C3H10T1/2 cells at the transcriptional and post-translational levels [40]. Moreover, PRMT1 prevents excessive degradation of triglycerides by WAT by limiting AMPK-mediated lipid catabolism [41]. We reported for the first time that PRMT1 knockdown restored fatty acid metabolism in TECs, thus improving energy homeostasis and reducing lipid accumulation and kidney injury. Further results support the potential renoprotective effect of lipid-lowering agents against calcium oxalate crystal-induced injury. To further evaluate the therapeutic potential of modulating lipid metabolism, we administered atorvastatin in the mouse model of CaOx crystal-induced kidney injury. Atorvastatin treatment significantly ameliorated tubular damage and attenuated lipid accumulation. These findings, in line with several recent studies reporting the protective effects of statins in various kidney diseases [42–45], underscore the critical role of lipid metabolic dysregulation in CaOx-induced renal injury and suggest that pharmacological modulation of lipid metabolism may serve as a complementary strategy to targeting the PRMT1/UBE2m axis.

PRMT1, a key epigenetic modifying enzyme, is involved in various cellular processes, including gene transcriptional regulation, RNA metabolism, and cell signaling [46]. For example, PRMT1 overexpression is associated with increased arginine methylation and aberrant exon inclusion of SRSF1, which is essential for breast cancer cell growth [47]; PRMT1 suppresses the anti-tumor immune response via arginine methylation of cGAS [48]. Thus, it is considered a promising therapeutic target in cancer treatment. However, its function as a therapeutic target for nonneoplastic diseases remains largely elusive. Here, we verified for the first time that PRMT1 regulated fatty acid metabolism in TECs by regulating methylation of R169 of UBE2m.

Neddylation is a ubiquitin-like post-translational modification wherein the ubiquitin-like molecule NEDD8 is covalently conjugated to target substrates. This governs the activity and functionality of the modified proteins. This process is orchestrated by a sole E1 enzyme (NAE), two E2 enzymes (UBE2m and UBE2F), and multiple E3 enzymes [49, 50]. Numerous ubiquitin E3 ligases undergo neddylation upon conjugation with NEDD8, thereby modulating their activity [51–54]. This links ubiquitination and neddylation, indicating that neddylation affects the ubiquitination levels of target proteins affiliated with ubiquitin E3 ligases. Research has shown that NEDD4 expression in the kidney and liver is regulated by 20-hydroxyeicosatetraenoic acid through the neddylation modification pathway [55] and that Nedd4-2 activation regulates the ubiquitination modification of renal NBCe1 [56]. These studies highlight neddylation as a key modulator of E3 ligase function in renal physiology. Our study demonstrated that methylated UBE2m increases the neddylation level and protein stability of NEDD4. Since NEDD4 is a classical E3 ligase targeting PPARγ, this modification ultimately promotes PPARγ degradation and disrupts fatty acid homeostasis.

These findings combined revealed a previously undescribed signaling of the PRMT1-UBE2m-NEDD4-PPARγ axis that plays an essential role in fatty acid metabolism during CaOx crystals-induced kidney injury. It provides a new therapeutic target for preventing CaOx stone-induced kidney injury.

## Supplementary information

Supplementary materials

Full original blots

## Data Availability

Raw data from the RNA-seq analyses have been uploaded to the Sequence Read Archive (SRA) of NCBI (https://www.ncbi.nlm.nih.gov/sra) (Accession: PRJNA808816). Raw data from the scRNA-seq analyses have been uploaded to the Sequence Read Archive (SRA) of NCBI (Accession: PRJNA118693). Metabolomic and proteomic raw data have been uploaded to MetaboLights (https://www.ebi.ac.uk/metabolights) (Accession: MTBLS11839). The raw datasets used and analyzed during the current study are available from the corresponding author upon request.
