# Supplementary material for: PRMT1-mediated methylation of UBE2m promoting calcium oxalate crystal-induced kidney injury by inhibiting fatty acid metabolism
Source: Cell Death Dis. 2025 Jul 31;16(1):579. doi: 10.1038/s41419-025-07888-3 (PMC12313907; doi:10.1038/s41419-025-07888-3)
Supplement: Supplementary file 1 — Supplementary materials [file 41419_2025_7888_MOESM1_ESM.docx]

**Supplementary Materials**

**Supplementary Figures**


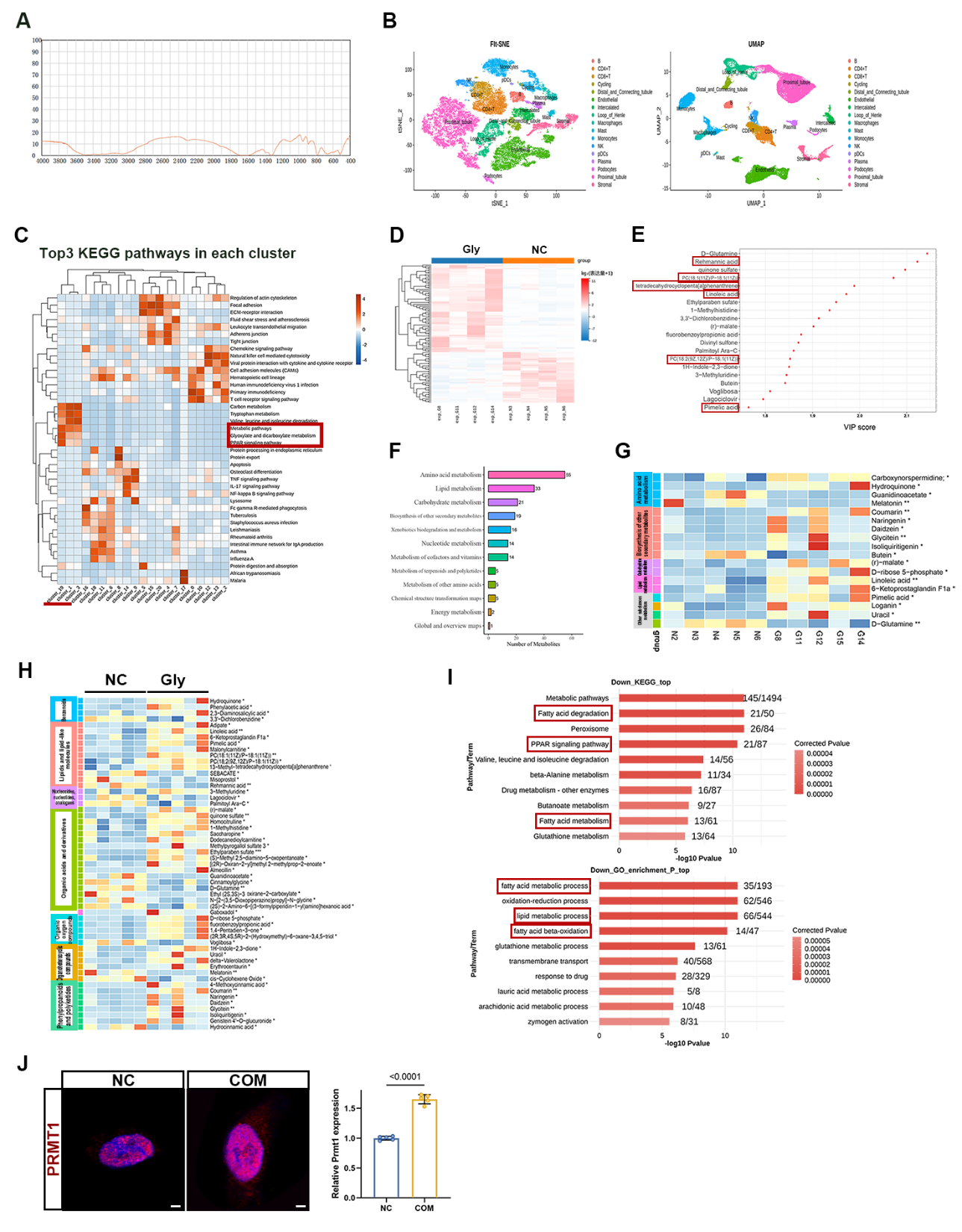


**Supplementary figure 1.** (A) Detection of stone components by infrared spectrum. (B) Cell-type clustering plot for unsupervised cell clustering. (C) KEGG enrichment analysis for genes significantly and specifically expressed in each cell cluster showed enriched pathways. (D) Clustering on differential metabolites between the NC and the Gly groups. (E) Differential metabolite VIP-score plot. (F) KEGG Pathway Barchart and X axis represents the number of metabolite annotations. (G) The heat map shows the differential metabolites of each pathway. (H) Heat maps showing differential metabolites between the NC and the Gly groups, assayed by untargeted metabolomics sequencing of kidney tissues. (I) KEGG and GO enrichment analysis of RNA sequencing data comparing DEGs between the NC (*n* = 3) and the KS group (*n* = 3). (J) Representative images of immunofluorescence staining of PRMT1 (red) in HK-2 cells with or without COM. Scale bars=50μm.

**
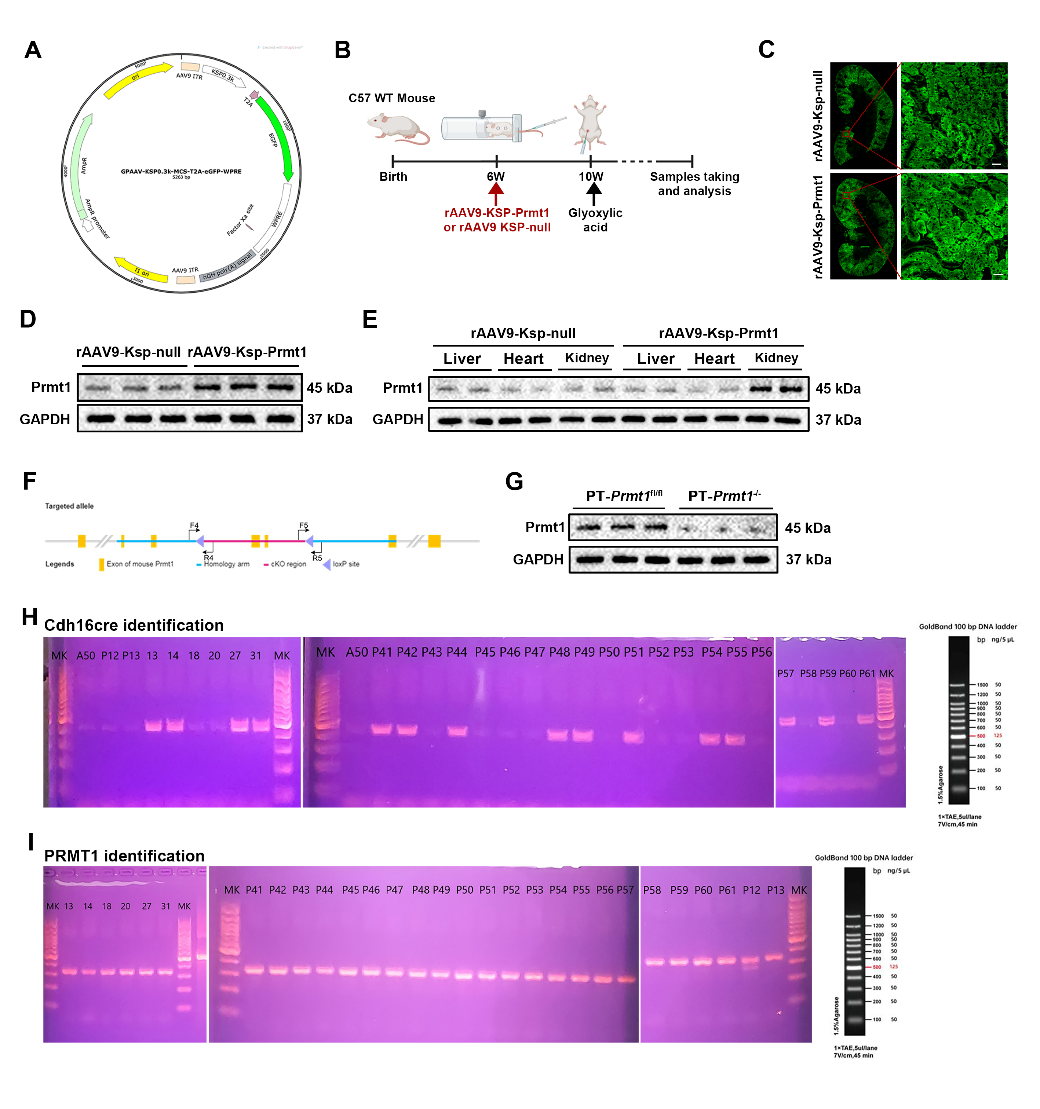
**

**Supplementary figure 2.** (A) Schematic diagram of plasmid composition of rAAV9. (B) Schematic diagram of construction procedure of mice specifically overexpressing PRMT1 in the tubular epithelial cells. (C) Representative images of immunofluorescence staining of EGFR1 (green) in kidneys of mice 4 weeks after injecting rAAV9-PRMT1 or rAAV9-null. Scale bars=50μm. (D) Representative western blot banding shows the expression levels of PRMT1of kidneys from the indicated groups in mice. (E) Representative images of western blot of PRMT1 from heart, liver or kidney tissue in indicated mice. (F) Schematic diagram of targeted allele. (G) Representative western blot banding shows the expression levels of PRMT1of kidneys from the indicated groups in mice. (I-J) Genotyping was performed by agarose gel electrophoresis.


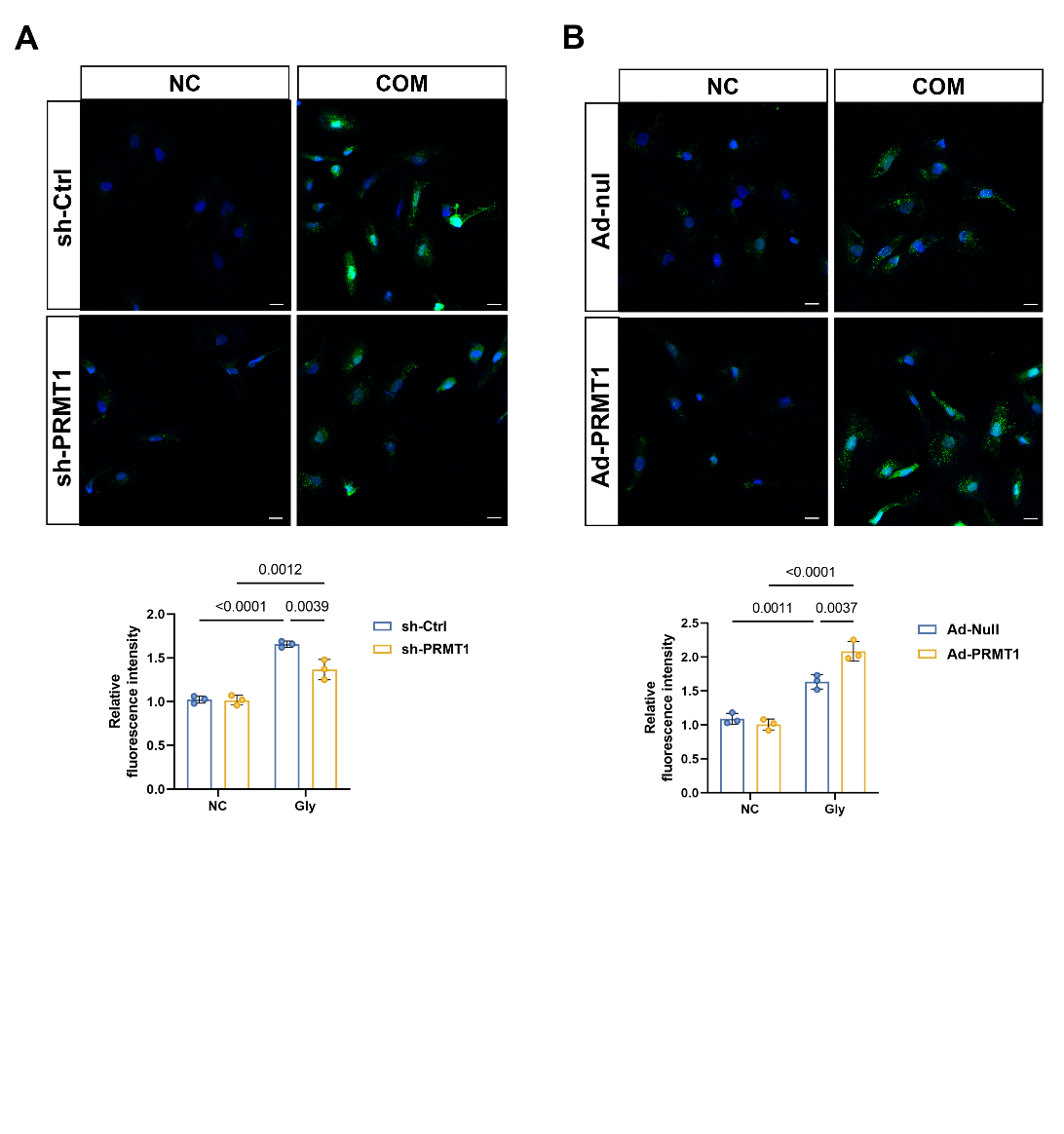


**Supplementary figure 3.** (A-B) Representative images and quantitative analysis of BODIPY staining from the indicated groups to detect lipid accumulation. Scale bars=50μm. Significance was assessed by 2-way ANOVA test. Data are shown as mean ± SD.

**
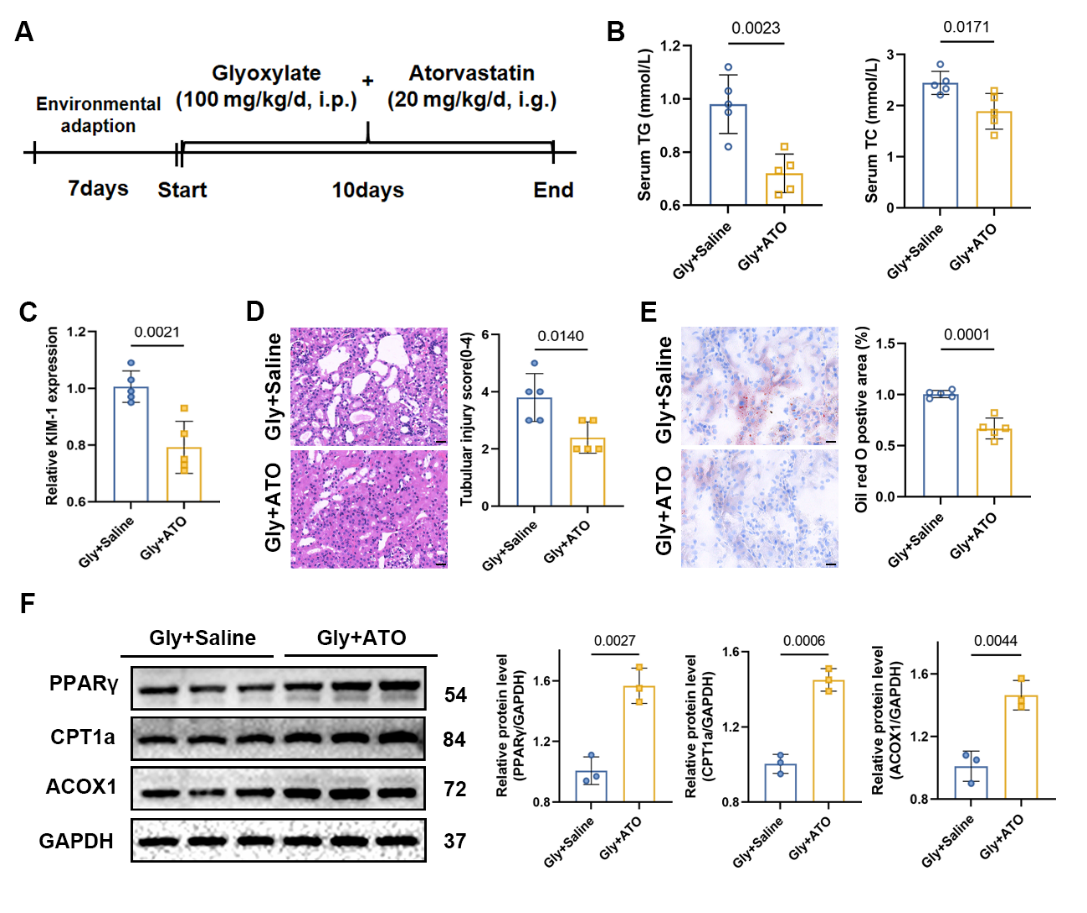
**

**Supplementary figure 4.** (A) Experimental design for atorvastatin prevention treatment on renal I/R injury. (B) Serum TG (triglyceride) and TC (cholesterol) levels of indicated groups. (C-E) Representative images of Vonkossa staining, H&E staining(D) and Oil Red staining(E) of CaOx‐injured kidneys from mice pre‐treated with vehicle or atorvastatin (20 mg/kg, i.g.) (400X); Scale bar = 50 μm. (F) Representative western blot bandings and quantitative analysis of the expression levels of ACOX1, CPT1a, CD36 and FATP2. Significance was assessed by t test. Data are shown as mean ± SD.

**
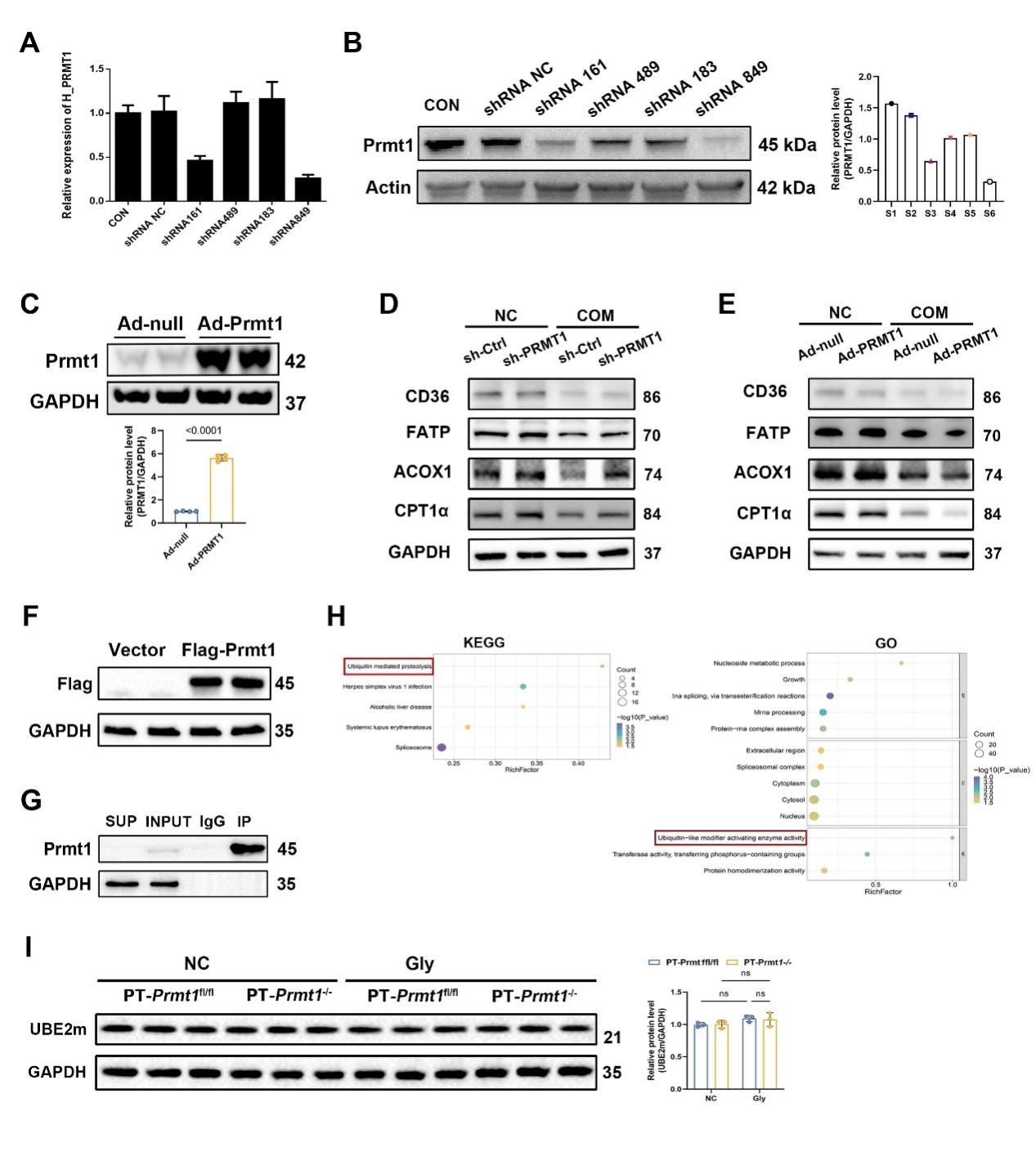
**

**Supplementary figure 5.** (A) RT-PCR was used to detect the knockdown efficiency of PRMT1 at different targets. (B) Representative western blot banding and quantitative analysis were used to detect the knockdown efficiency of PRMT1 at different targets. (C) Representative western blot banding was used to detect the PRMT1 overexpression efficiency after HK-2 cells were infected with Adv-PRMT1. (D-E) Representative western blot banding and quantitative analysis show the expression levels of CPT1α, ACOX1, CD36, and FATP2 in the indicated groups. (F) Representative western blot banding was used to detect the transfection efficiency of Flag-PRMT1 into HK-2 cells. (G) Representative western blot banding was used to verified IP efficiency. (H) GO and KEGG enrichment analysis of 99 interacting proteins (Fold Chang≥4) identified by MS. (I) Representative western blot banding and quantitative analysis show the expression levels of UBE2m in the indicated groups. Significance was assessed by 2-way ANOVA test. Data are shown as mean ± SD.

**
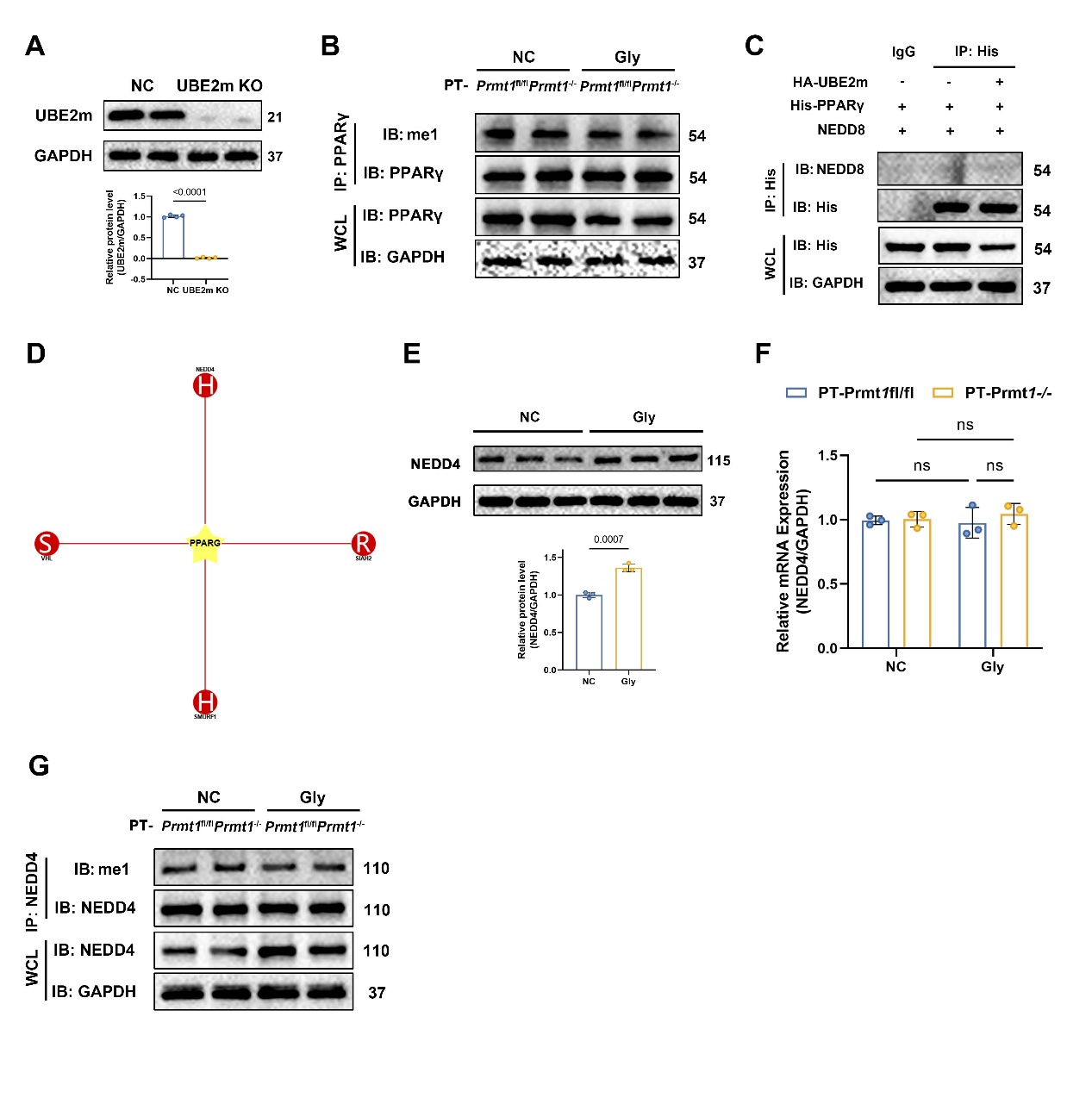
**

**Supplementary figure 6.** (A) Representative western blot banding and quantitative analysis were used to detect the UBE2m knockout efficiency in HK-2 cells. (B) Detection of endogenous me1 levels of PPARγ in the indicated groups in mice. (C) Detection of exogenous neddylation levels of PPARγ in the indicated groups in mice. (D) UbiBrowser 2.0 database indicated the classical E3 ubiquitin ligases of PPARγ. (E) Representative western blot banding and quantitative analysis show the expression levels of NEDD4 in the NC and the Gly groups. (F) qPCR results show the RNA levels of NEDD4 from the indicated groups. (G) Detection of endogenous me1 levels of NEDD4 from the indicated groups in mice. Significance was assessed by 2-way ANOVA or t test. Data are shown as mean ± SD.

**
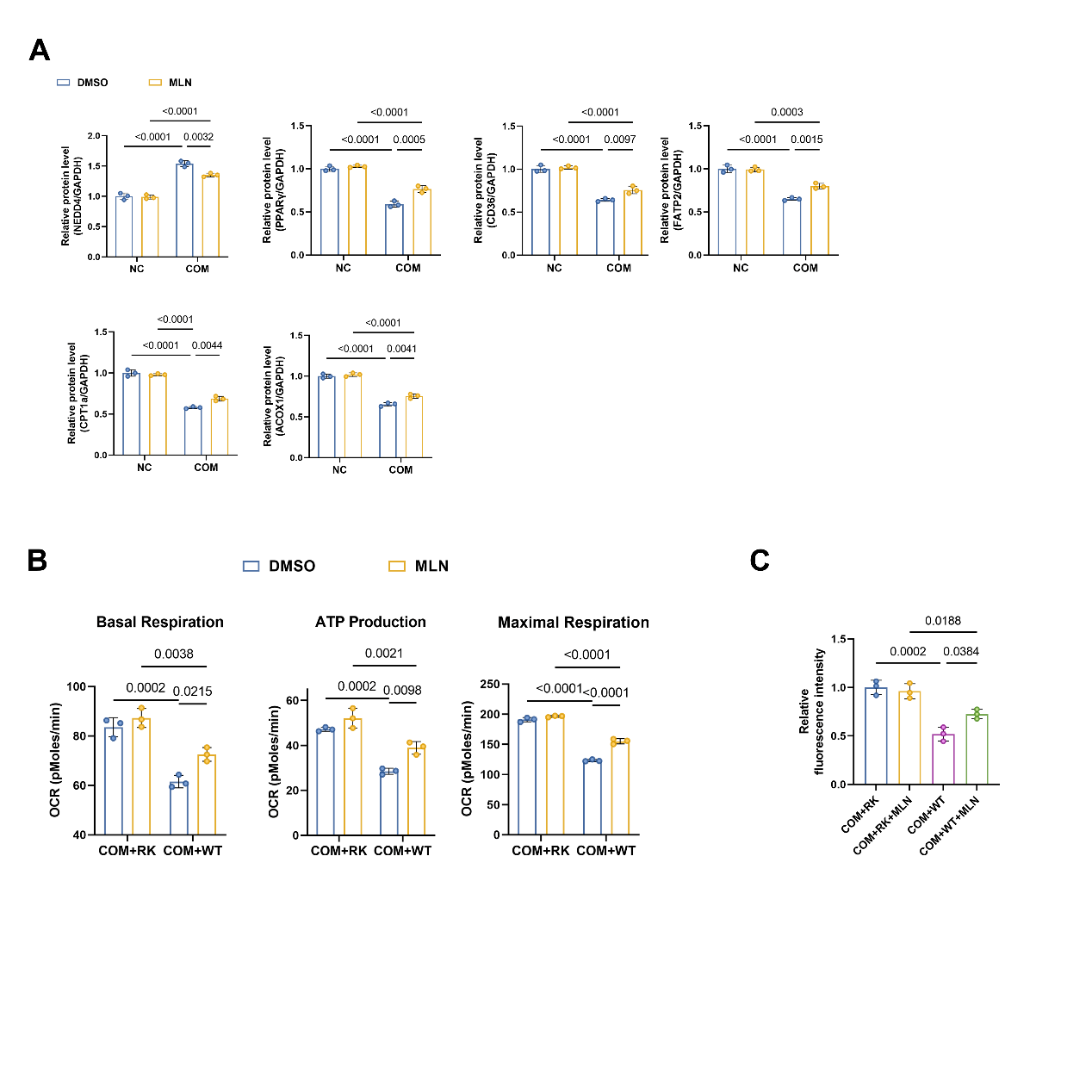
**

**Supplementary figure 7.** (A) Quantitative analysis of WB bands in Figure7.J shows the expression levels of NEDD4, PPARγ, CD36, FATP2, CPT1α, and ACOX1 in the indicated groups in HK-2 cells. (B) Basal respiration, ATP production-coupled respiration, and maximal respiration were quantified. (C) Quantitative analysis of fluorescence intensity in Figure7.L shows the level of FAO.

| **Number** | **Age, yrs**  **(Mean±SD)** | **Number**  **（Male/Female）** | **eGFR**  **(ml/min)** |
| --- | --- | --- | --- |
| KS(n=8) | | | |
| 1 | 49.1±8.0 | 5/3 | 10.1 |
| 2 |  |  | 9.1 |
| 3 |  |  | 9.3 |
| 4 |  |  | 12.5 |
| 5 |  |  | 11 |
| 6 |  |  | 13.6 |
| 5 |  |  | 15.8 |
| 8 |  |  | 15.4 |
| Normal(n=8) | | | |
| 1 | 59.9±6.1 | 5/3 | 95.7 |
| 2 |  |  | 110.4 |
| 3 |  |  | 87.5 |
| 4 |  |  | 98.7 |
| 5 |  |  | 105 |
| 6 |  |  | 80.2 |
| 5 |  |  | 102.3 |
| 8 |  |  | 100.2 |

**Table S1: Clinical characteristics of the patients.**

**Tables. S2: Protein sequences of PRMT1, UBE2m and GST**

| **Protein** | **Sequence** |
| --- | --- |
| GST-PRMT1 | MEVSCGQAES SEKPNAEDMT SKDYYFDSYA HFGIHEEMLK DEVRTLTYRN SMFHNRHLFK DKVVLDVGSG TGILCMFAAK AGARKVIGIE CSSISDYAVK IVKANKLDHV VTIIKGKVEE VELPVEKVDI IISEWMGYCL FYESMLNTVL YARDKWLAPD GLIFPDRATL YVTAIEDRQY KDYKIHWWEN VYGFDMSCIK DVAIKEPLVD VVDPKQLVTN ACLIKEVDIY TVKVEDLTFT SPFCLQVKRN DYVHALVAYF NIEFTRCHKR TGFSTSPESP YTHWKQTVFY MEDYLTVKTG EEIFGTIGMR PNAKNNRDLD FTIDLDFKGQ LCELSCSTDY RMR |
| His-UBE2m | MGSSHHHHHH SSGLVPRGSH MGSHMIKLFS LKQQKKEEES AGGTKGSSKK ASAAQLRIQK DINELNLPKT CDISFSDPDD LLNFKLVICP DEGFYKSGKF VFSFKVGQGY PHDPPKVKCE TMVYHPNIDL EGNVCLNILR EDWKPVLTIN SIIYGLQYLF LEPNPEDPLN KEAAEVLQNN RRLFEQNVQR SMRGGYIGST YFERCLK |
| GST | MSPILGYWKI KGLVQPTRLL LEYLEEKYEE HLYERDEGDK WRNKKFELGL EFPNLPYYID GDVKLTQSMA IIRYIADKHN MLGGCPKERA EISMLEGAVL DIRYGVSRIA YSKDFETLKV DFLSKLPEML KMFEDRLCHK TYLNGDHVTH PDFMLYDALD VVLYMDPMCL DAFPKLVCFK KRIEAIPQID KYLKSSKYIA WPLQGWQATF GGGDHPPKSD LVPRGSPGIH RD |

**Tables. S3: Primers for qPCR**

| **Primer name** | **Forward primer** | **Reverse primer** |
| --- | --- | --- |
| Human-PRMT1 | CTCCTACGCACACTTTGGCA | GTTGCGGTAAGTGAGGGTGC |
| Mouse-PRMT1 | GAGCTTTTGGAGGCCTAGGGAC | GAGCTTTTGGAGGCCTAGGGAC |
| Mouse-UBE2m | AACCTGCCCAAGACGTGTG | AGCTGAATACAAACTTGCCACT |
| Mouse-FASN | GGAGGTGGTGATAGCCGGTAT | TGGGTAATCCATAGAGCCCAG |
| Mouse-Me1 | GTCGTGCATCTCTCACAGAAG | TGAGGGCAGTTGGTTTTATCTTT |
| Mouse-SCD-1 | TTCTTGCGATACACTCTGGTGC | CGGGATTGAATGTTCTTGTCGT |
| Mouse-CD36 | AGATGACGTGGCAAAGAACAG | CCTTGGCTAGATAACGAACTCTG |
| Mouse-Slc27a2 | TCCTCCAAGATGTGCGGTACT | TAGGTGAGCGTCTCGTCTCG |
| Mouse-Acsl1 | TGCCAGAGCTGATTGACATTC | GGCATACCAGAAGGTGGTGAG |
| Mouse-CPT1α | CTCCGCCTGAGCCATGAAG | CACCAGTGATGATGCCATTCT |
| Mouse-CPT2 | CAGCACAGCATCGTACCCA | TCCCAATGCCGTTCTCAAAAT |
| Mouse-ACOX1 | TAACTTCCTCACTCGAAGCCA | AGTTCCATGACCCATCTCTGTC |
| Mouse-ACOX3 | ACCGGAAGAAAAAGACAGTGC | GAGGCTCTTGCTCGGTAGG |
| Mouse-PPARγ | TCGCTGATGCACTGCCTATG | GAGAGGTCCACAGAGCTGATT |
| Mouse-NEDD4 | TCGGAGGACGAGGTATGGG | GGTACGGATCAGCAGTGAACA |
| Human-GAPDH | GGAGCGAGATCCCTCCAAAAT | GGCTGTTGTCATACTTCTCATGG |
| Mouse-GAPDH | AGGTCGGTGTGAACGGATTTG | TGTAGACCATGTAGTTGAGGTCA |

**Tables. S4: Primers for mice identification**

| **Primer name** | **Forward primer** | **Reverse primer** |
| --- | --- | --- |
| PRMT1 | GAGCTTTTGGAGGCCTAGGGAC | GAGCTTTTGGAGGCCTAGGGAC |
| Cdh16-Cre | GCAGATCTGGCTCTCCAAAG | AGGCAAATTTTGGTGTACGG |

**Tables. S5: shRNA for gene knockdown**

| **Gene name** | **Sequence** |  |
| --- | --- | --- |
| UBE2m-shRNA | 5’-GCGGATCCAGAAGGACATAAA-3’ | Genomeditech |
| NEDD4-shRNA | 5’-CGCCTTGACTTACCTCCATAT-3’ | Genomeditech |
| PRMT1-shRNA849 | 5’-CCTGGTGGCCTACTTCAACAT-3’ | Genomeditech |

**Tables. S6: sgRNA for gene knockout**

| **Gene name** | **Sequence** |  |
| --- | --- | --- |
| Ube2m sgRNA-1 | 5’- CACCCCAACATTGACCTCG-3’ | MERCK |
