# Supplementary material for: PRMT1-mediated methylation of UBE2m promoting calcium oxalate crystal-induced kidney injury by inhibiting fatty acid metabolism
Source: Cell Death Dis. 2025 Jul 31;16(1):579. doi: 10.1038/s41419-025-07888-3 (PMC12313907; doi:10.1038/s41419-025-07888-3)

Figure1.D

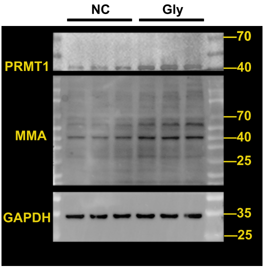

Figure2.D

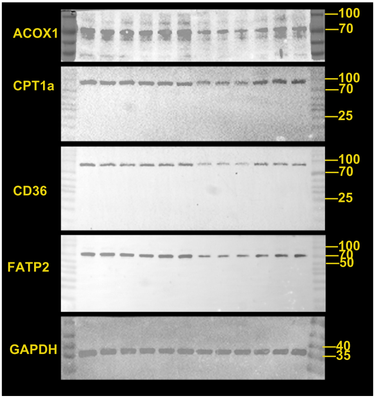

Figure3.D

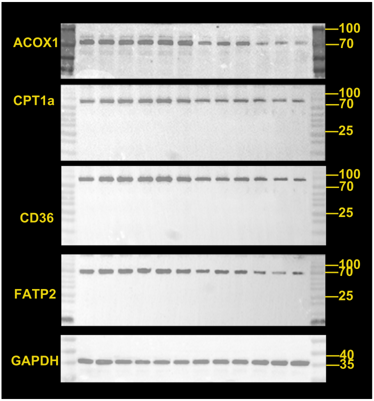

Figure4.F

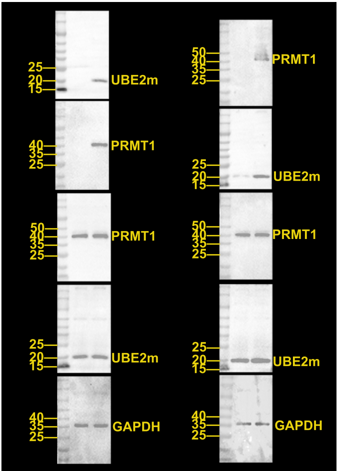

Figure4.G

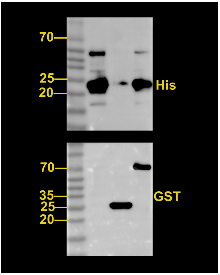

Figure4.H

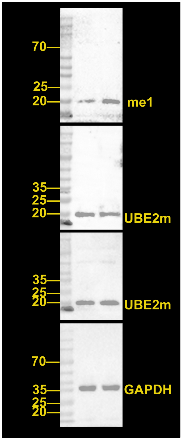

Figure4.J

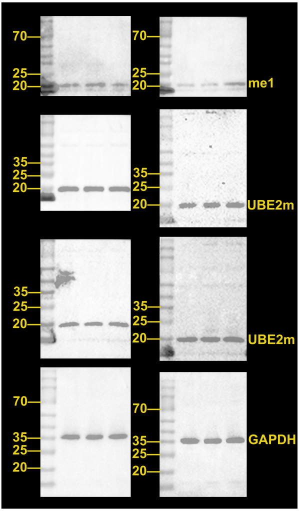

Figure5.D

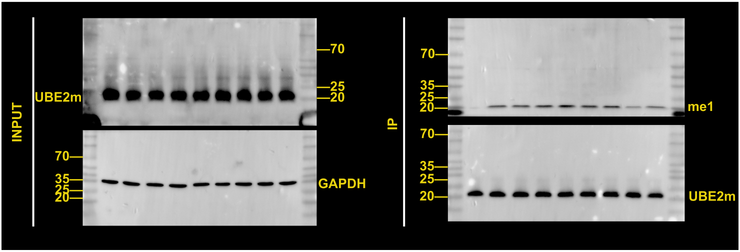

Figure5.E

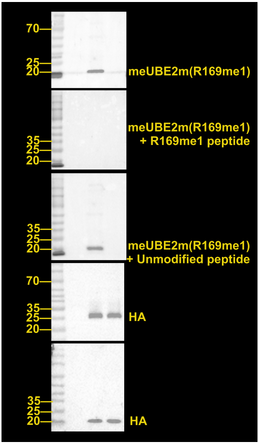

Figure5.G

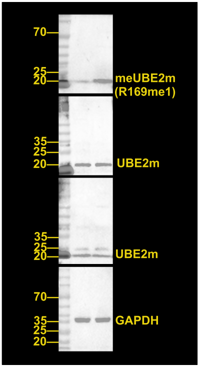

Figure5.H

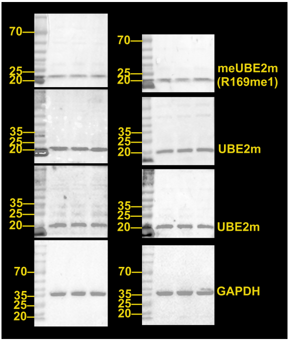

Figure5.I

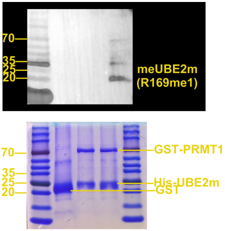

Figure6.B

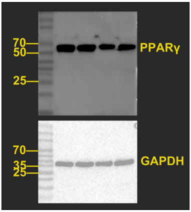

Figure6.C

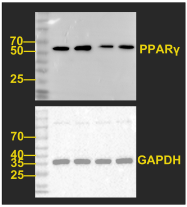

Figure6.D

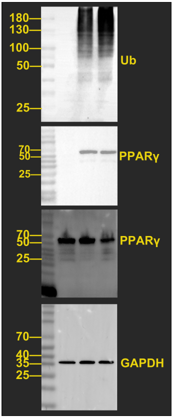

Figure6.E

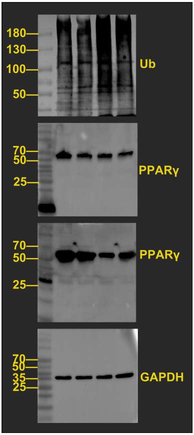

Figure6.F

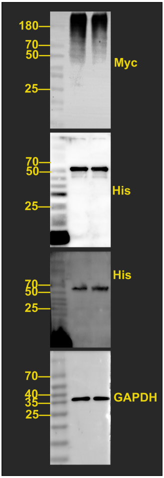

Figure6.G

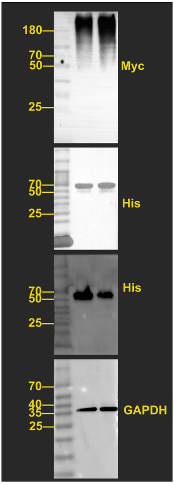

Figure6.H

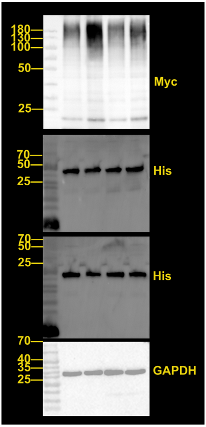

Figure6.I

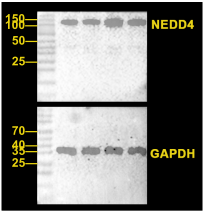

Figure6.J

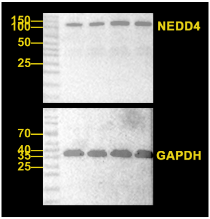

Figure6.K

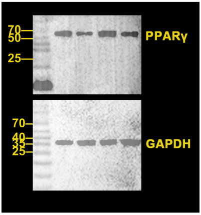

Figure6.M

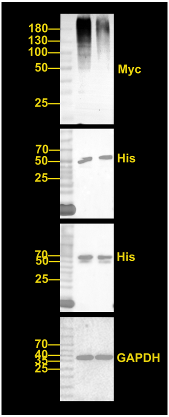

Figure6.N

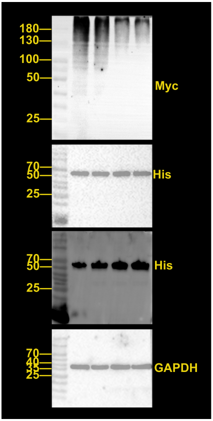

Figure7.A

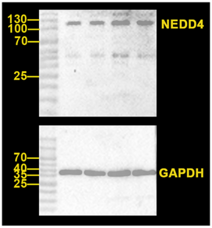

Figure7.B

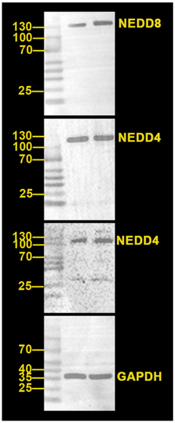

Figure7.C

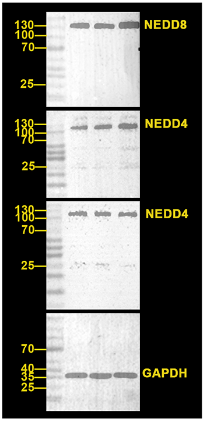

Figure7.D

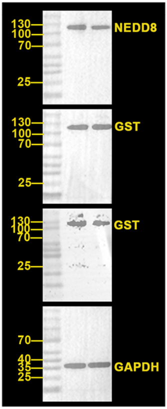

Figure7.E

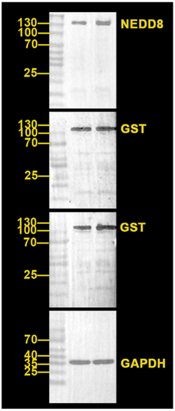

Figure7.F

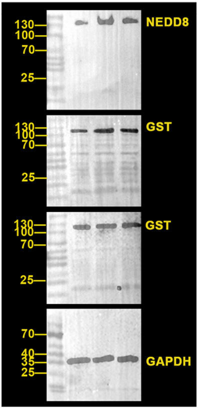

Figure7.G

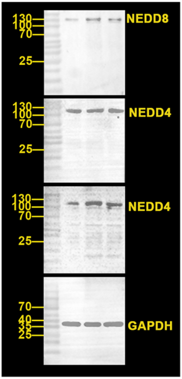

Figure7.I

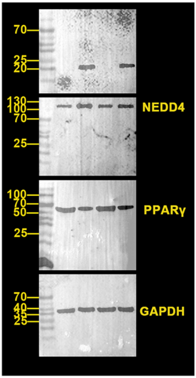

Figure7.J

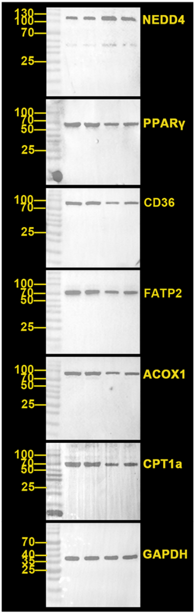

FigureS2.D

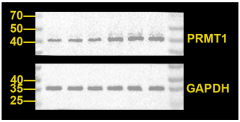

FigureS2.E

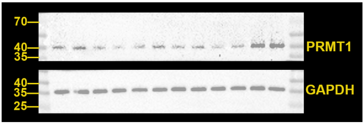

FigureS2.H

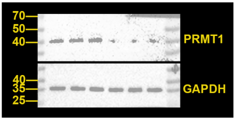

FigureS5.D

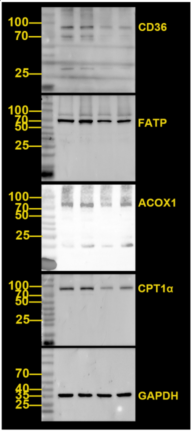

FigureS5.E

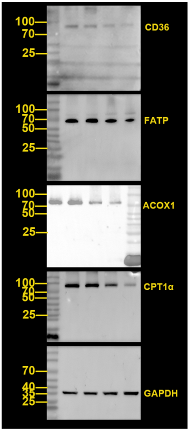

FigureS5.F

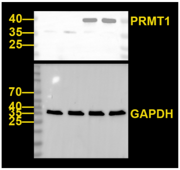

FigureS5.G

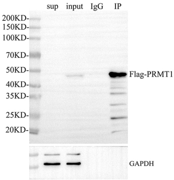

FigureS5.I

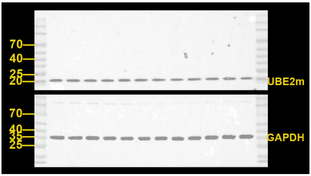

FigureS6.A

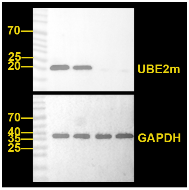

FigureS6.B

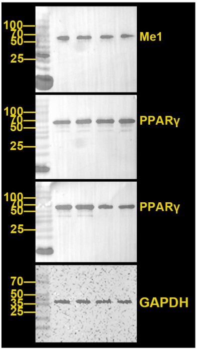

FigureS6.C

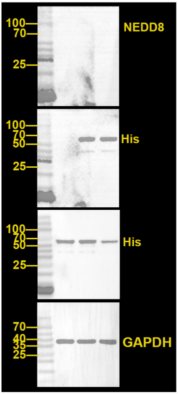

FigureS6.E

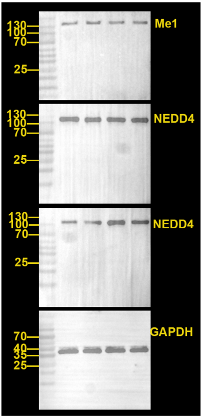

FigureS6.G

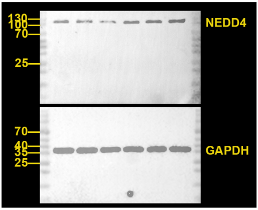

Supplement: Supplementary file 2 — Full original blots [file 41419_2025_7888_MOESM2_ESM.pdf]
